# *MYCN* Drives a Tumor Immunosuppressive Environment Which Impacts Survival in Neuroblastoma

**DOI:** 10.3389/fonc.2021.625207

**Published:** 2021-02-25

**Authors:** Salvatore Raieli, Daniele Di Renzo, Silvia Lampis, Camilla Amadesi, Luca Montemurro, Andrea Pession, Patrizia Hrelia, Matthias Fischer, Roberto Tonelli

**Affiliations:** ^1^ R&D Department, BIOGENERA SpA, Bologna, Italy; ^2^ Department of Pharmacy and Biotechnologies, University of Bologna, Bologna, Italy; ^3^ Pediatric Unit, IRCCS Azienda Ospedaliero-Universitaria di Bologna, Bologna, Italy; ^4^ Department of Experimental Pediatric Oncology, Medical Faculty, University Children’s Hospital of Cologne, Cologne, Germany; ^5^ Center for Molecular Medicine Cologne (CMMC), University of Cologne, Cologne, Germany

**Keywords:** *MYCN*, immune system, neuroblastoma, immune signature, immune network, anti-*MYCN* antigene PNA, *MYCN* blocking

## Abstract

A wide range of malignancies presents *MYCN* amplification (MNA) or dysregulation. *MYCN* is associated with poor prognosis and its over-expression leads to several dysregulations including metabolic reprogramming, mitochondria alteration, and cancer stem cell phenotype. Some hints suggest that *MYCN* overexpression leads to cancer immune-escape. However, this relationship presents various open questions. Our work investigated in details the relationship of *MYCN* with the immune system, finding a correlated immune-suppressive phenotype in neuroblastoma (NB) and different cancers where *MYCN* is up-regulated. We found a downregulated Th1-lymphocytes/M1-Macrophages axis and upregulated Th2-lymphocytes/M2-macrophages in MNA NB patients. Moreover, we unveiled a complex immune network orchestrated by N-Myc and we identified 16 genes modules associated to MNA NB. We also identified a *MYCN*-associated immune signature that has a prognostic value in NB and recapitulates clinical features. Our signature also discriminates patients with poor survival in non-MNA NB patients where *MYCN* expression is not discriminative. Finally, we showed that targeted inhibition of *MYCN* by BGA002 (anti-*MYCN* antigene PNA) is able to restore NK sensibility in *MYCN*-expressing NB cells. Overall, our study unveils a *MYCN-*driven immune network in NB and shows a therapeutic option to restore sensibility to immune cells.

## Introduction

*MYCN* is a transcription factor member of the *MYC* proto-oncogene family involved in nervous system development during embryogenesis (1). *MYCN* regulates different fundamental cellular processes including cell cycle, apoptosis, mitochondria dysfunction, and metabolism (2, 3). Indeed, *MYCN* expression deregulation is linked to a wide range of human tumors (4). *MYCN* overexpression is generally associated to poor prognosis, driving the cancer cells to a stem cell like phenotype, promoting growth, angiogenesis, and metastasis (5, 6).

Considering the *MYCN* restricted expression in embryogenesis and it is impact in cancer (7), N-Myc is a promising target. However, small molecule approaches to specifically target the N-Myc protein resulted inconsistent. Thus, different other approaches have been developed to downregulate N-Myc or its associated pathways (8). Among these strategies, anti-*MYCN* antigene oligonucleotide PNA showed the ability to specifically block *MYCN* expression in a sustained way (3, 9, 10), resulting in an anti-cancer effect.

*MYCN* amplification (MNA) is established as a major driver of Neuroblastoma (NB) (characterizing 50% of the high-risk group) (11–13). NB is currently the most common and deadly pediatric solid cancer (representing 6–8% of solid tumors in childhood) (14). Current therapy includes chemotherapy, radiotherapy, surgery, and stem cell transplantation, besides the severe side effects still many patients undergo relapse and progression (15–17).

Immune evasion plays a fundamental role in the development and progression of cancers and is driven by the tumor microenvironment remodeling by cancer cells (18). Different studies showed that NB presents the capacity to evade and to harness the immune system to favor metastasis and progression. In this view, tumor infiltrating lymphocytes (CD4+ and CD8+ T-cells) and natural killers (NKs) are favorable associated with the outcome while T regulatory cells and macrophages are associated with poor prognosis (19–21). NB cells can express checkpoint inhibitors or other molecules capable to interact with the immune system such as PD-L1, MIF, chemokines, release of microRNAs to microenvironment cells, suggesting the potential impact of checkpoint inhibitors and immune-therapy also in this tumor (22–25).

However, while anti-GD2 therapy and chimeric antigen receptor (CAR) T cells showed some promising results in non-MNA NB patients, the checkpoint inhibitors have not shown the same success in improving the survival in NB, as in other solid tumors. These poor results can be linked to different factors as low MHC-I expression, low presence of neoantigens, immunosuppressive environment (15, 26–28). Moreover, N-Myc could play a role in the development of this immunosuppressive microenvironment, as MNA is associated to down-regulation of MHC-I expression in NB and to inhibition of the interferon pathway and to PD-L1 expression (29–32). Indeed, MNA-NB still shows a poor outcome and need a missing specific therapy.

In this context, the tremendous amount of interactions between NB cells and the tumor microenvironment leads to a high complexity, leaving different open questions on how NB harness the immune system to sustain its growth and which factors have a dominant role or have context dependent functions. No studies systematically analyzed immune cell infiltration and their molecular interactions or broadly investigate *MYCN* impact on the immune system. Indeed, the NB immunity and the role of *MYCN* in the immunosuppression are still a field of investigation (33).

## Materials and Methods

### Patient Gene Expression Profiles

The NB dataset (accession: E-MTAB-1781) and the small cell lung cancer (SCLC) (accession: E-MTAB-1999) datasets were downloaded from ArrayExpress, (http://www.ebi.ac.uk/arrayexpress) and processed using the quantile algorithm in *limma*. Another NB dataset and Wilms’ dataset were downloaded from TARGET data portal (https://ocg.cancer.gov/programs/target; data freely accessible). From NCBI GEO DataSets (https://www.ncbi.nlm.nih.gov/gds) were downloaded the following datasets: retinoblastoma (accession: GSE59983), rhabdomyosarcoma (accession: GSE114621), T acute lymphoblastic leukemia (T-ALL), and acute myeloid leukemia (AML) dataset (accession: GSE13159, as defined in the dataset meta-data) and for the T-helper lymphocyte (Th) profiles (accession: GSE107011). Another SCLC dataset was retrieved from the supplementary of the article (PMID: 26168399) (34). In the article the different cohorts are referred as following: NB1: E-MTAB-1781, NB2: TARGET NB, Wilms: TARGET Wilms, SCLC1: E-MTAB-1999, SCLC2: PMID 26168399, RB: GSE59983, rhabdomyosarcoma (RMS): GSE114621, T-ALL: GSE13159, AML: GSE13159. The replicate probes within the array were replaced by their average before being scaled. Pearson correlation between *MYCN*, *MYC*, and other genes was calculated with R software. The differential expressed genes between *MYCN*-amplified (MNA) patients and non-MNA patients in the NB datasets (E-MTAB-1781, TARGET dataset) were obtained using the *limma* package algorithm (clinical information was retrieved from the dataset meta-data, patient with an unknown MNA status were removed).

### Pathway Analysis

Correlated genes with *MYCN* or *MYC* in each dataset were used as ranked gene list to identify enriched pathway through Gene Set Enrichment Analysis (GSEA) (35). We used Gene Ontology (biological process, cellular component, molecular function, C5 from Molecular Signatures Database v7.0), and GSEA software (V. 4.02). We used the differential expressed genes to conduct the pathway enrichment as described above. Graphic representation was performed with R software. Additional data can be found in **Supplementary Tables 1–3**.

### Immune Cell Fraction Estimation Analysis

We used CIBERSOFT tool (Cell type Identification By Estimating Relative Subsets Of known RNA Transcripts) as described in the developer instruction (36). NB expression datasets (E-MTAB-1781, TARGET dataset) were used as mixture file input and were performed 1,000 permutations. We used the LM22 gene signature matrix, an available validated signature for 22 human hematopoietic cell phenotypes. Additionally, we derived from GSE107011 the signature for the Th profile and to generate a Th gene signature matrix (we considered Th1, Th2, Th17, T-regulatory, T follicular helper subsets). The Th signature was input in CIBERSOFT and performed 1,000 permutations. Graphic representation and statistical analysis were performed with R software.

### Immune Interaction Network

Protein interactions were retrieved from String database, we selected interactions with a score higher than 400. Protein localization was downloaded from Human Protein Atlas (37). We considered as cell surface proteins only proteins which the approved and supported locations included one of the following terms: cell junctions, focal adhesion sites, plasma membrane. Immune population protein expression were obtained from data supplementary (38). We considered as expressed for each immune subpopulation only proteins with a normalized score higher than 1.5 and only if present in the cell surface. The immune network in MNA and non-MNA patients were established filtering the immune surface proteins present in the differential expressed genes (in order to capture weak interactions, we considered log fold change of 0.5 to assign to MNA or non-MNA). The protein-protein interactions were used to build the circular plots. *MYCN* knock out genes were downloaded by KnockTF (39), we considered log fold change of 1 to assign a gene to the *MYCN* positive or negative regulated list. The previous lists were filtered for the subcellular position. The obtained genes were paired with their possible interactors on the immune population to build the circular plots.

### WGCNA Module Analysis and Transcriptional Regulator

Immune population protein expression was retrieved as described above. Patient gene expression profiles (GEP) from E-MTAB-1781 were filtered for this list. WGCNA (40) was performed using the WGCNA package (41) and changing the standard parameters: power of 8, signed network, and a minimum module size of 20. The algorithm assigned the 6,641 filtered genes to 16 modules (2,472 were not assigned to any module, the full list is present in **Supplementary Table 4**). Module similarity was conducted calculating Pearson correlation between module eigengenes. Cell populations were grouped in (CD4+ T-cells, CD8+ T-cells, antigen processing cells, B-cells, NK cells) and calculated the number of proteins present in each module. Graph network was building using the iGraph package. For the heatmap, we calculated the number of proteins present in each module for each immune population and then normalized (z-score). We also calculated the average of the module eigengenes for MNA and non-MNA patient groups and then we normalized (z-score). Pathway enrichment was conducted for each module using anRichment package, the results are present in **Supplementary Table 5**. Gene modules were used to infer transcriptional regulators, we then clustered the obtained regulators in three clusters. Patient expression profiles were also clustered according to the cluster regulators. This procedure is also described in details in **Supplementary Methods** and **Supplementary Tables 6**-**8**.

### MYCN Immune Score

Immune genes retrieved from Gene Ontology (GO) and literature. Patient gene expression profiles (GEP) from E-MTAB-1781 and TARGET were filtered for this list (gene list is present in **Supplementary Table 9**). We build a logistic regression model to identify which immune genes where associated to MNA *versus* not MNA patients. The model was cross-validated 50-fold using E-MTAB-1781 as training set (80% of observation at each run) and using TARGET dataset as test set. We used L1 penalization (C = 0.1) and SAGE solver. We selected the weights for each gene and averaged (we filtered all zero weights). We selected two different vector weights associated with MYCN, positive weight vector (associated with MNA) and negative weight vector (associated with non-MNA). The two vectors were normalized subtracting the minimum and dividing by the range:

$${\hat{W}}_{i}^{PM}=\frac{W_{i}^{PM}-\text{min}\left(W^{PM}\right)}{\max\left(W^{PM}\right)-\text{min}\left(W^{PM}\right)}$$

$${\hat{W}}_{i}^{NM}=\frac{W_{i}^{NM}-\text{min}\left(W^{NM}\right)}{\max\left(W^{NM}\right)-\text{min}\left(W^{NM}\right)}$$

Through univariate cox regression we selected genes significantly associated to the prognosis (we used the same gene list used for the logistic regression model). The obtained p-value was correct with the Bonferroni correction (list of significant genes obtained through univariate cox regression is present in **Supplementary Table 10**). We then used multivariate Cox regression analysis on the obtained genes, we used a Lasso penalization to select genes associated to the prognosis [we used Penalized R package (42) and selecting lambda1 parameter equal to 0.25]. We selected two different vector weights associated with the survival, positive weight vector (associated with hazard) and negative weight vector (associated with reduction in hazard). The two vectors were normalized subtracting the minimum and dividing by the range:

$${\hat{W}}_{i}^{PC}=\frac{W_{i}^{PC}-\text{min}\left(W^{PC}\right)}{\max\left(W^{PC}\right)-\text{min}\left(W^{PC}\right)}$$

$${\hat{W}}_{i}^{NC}=\frac{W_{i}^{NC}-\text{min}\left(W^{NC}\right)}{\max\left(W^{NC}\right)-\text{min}\left(W^{NC}\right)}$$

We selected the genes in common between the two vectors and normalized weight were calculated as the sum of the normalized vector for *MYCN* and Cox model. We then build a positive and a negative immune score for each dataset (-MTAB-1781 and TARGET) multiplying each gene x (log2 expression) for each normalized weight (weights are listed in **Supplementary Table 11**).

$$imm^{P}=\;\frac{1}{n}\sum_{i=1}^{n}x_{i}({\hat{W}}_{i}^{PM}+{\hat{W}}_{i}^{PM})$$

$$imm^{N}=\;\frac{1}{n}\sum_{i=1}^{n}x_{i}\left({\hat{W}}_{i}^{NM}+{\hat{W}}_{i}^{NC}\right)$$

We defined the *MYCN* immune score as the ratio between imm^p^ and imm^n^ for each patient GEP. We download Neuroblastoma (last 10 years) and *MYCN* abstracts querying PubMed (details are present in the **Supplementary Methods**) to identify genes of the signature present in literature. Patient were stratified according their positive or negative normalized *MYCN* immune score (low enriched was defined as z score lower than −0.5, medium enriched comprised between −0.5 and 1, high enriched z score higher than 1). Clinical information was retrieved within the dataset (**Supplementary Tables 12** and **13**). Uniform Manifold Approximation and Projection (UMAP) was computed with a minimum distance of 0.5, considering 30 local neighbors and selecting the Euclidean distance as metric (43).

### Statistical Analysis and Software

Analysis were conducted in R (RStudio) and Python (Anaconda release). The following libraries from Python (version 3.7) were used: Scikit-learn, Matplotlib, matplotlib.pyplot, Pandas, UMAP, numpy. The following libraries from R (version 3.5) were used for analysis and graphs: ggplot2, dplyr, data.table, tydr, survival, survminer, wordcloud, WGCNA, circlize, iGraph, anRichment, stringr.

### Cell Lines and Treatment

The cell lines used in this study were obtained in 2020 and kept in culture for 30 days and seven passages at maximum. Mycoplasma detection was conducted with LookOut Mycoplasma PCR Detection Kit (Sigma-Aldrich). Additional details about the cell line used in this study can be found in **Supplementary Table 14**. Cell lines treatment with BGA002 and quantitative real-time PCR were conducted as described in (3). List of the primers used in this study can be found in **Supplementary Table 15**. Results have been analyzed in Prism software version 6 (GraphPad).

### Neuroblastoma Cell Lines and Natural Killers Co-Culture

Kelly-luc cell-line (Kelly NB cell line transfected with luciferase gene) was generated as described in (3). Kelly-luc has been treated with 2.5 µM of BGA002 for 12 h in Opti-MEM. PBMC from healthy donors has been isolated through Ficoll protocol and resuspended in Opti-MEM. NK cells have been isolated using Human NK Cell Enrichment Set–DM (cat no. 557987, BD Bioscience). NB-NK co-culture has been performed in Opti-MEM for 4 h After adding D-luciferine and lysis buffer we measured luminescence Infinite F200 Tecan. Results have been analyzed in Prism software version 6 (GraphPad).

## Results

### MYCN Is Associated With Immune Repression and a Th2-Lymphocytes/M2-Macrophages Axis Upregulation

**Figure 1:**
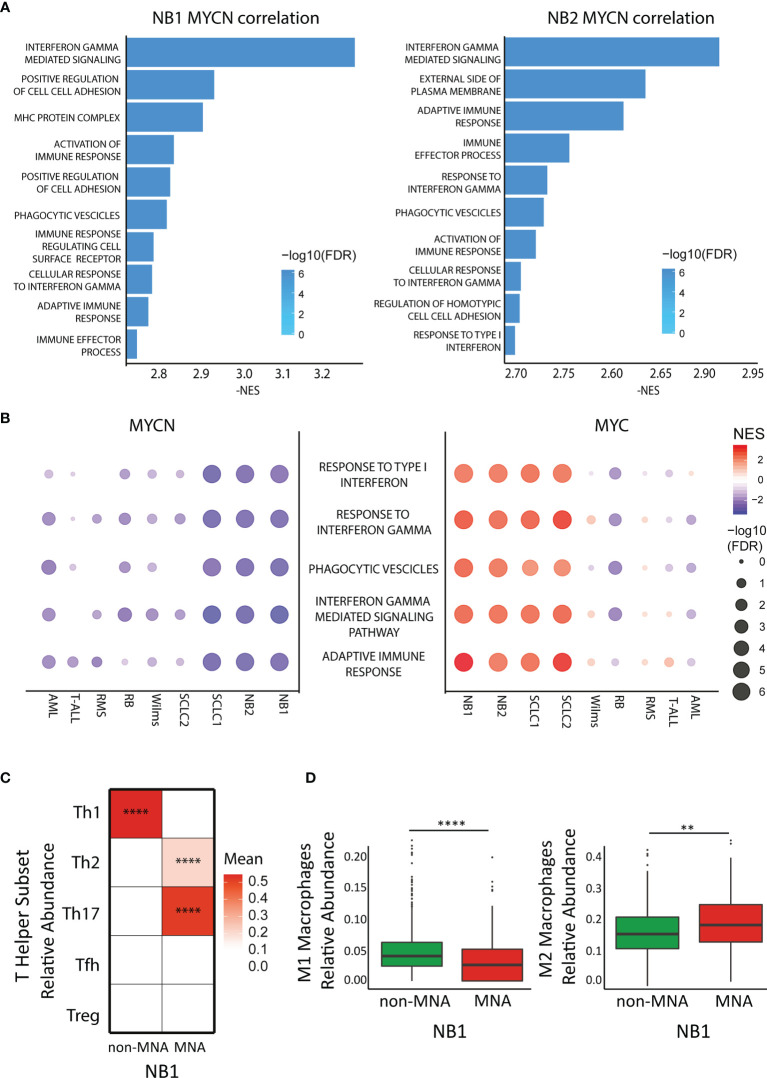
*MYCN* but not *MYC* anticorrelates with immune pathways in cancers. **(A)** Bar plot represents Gene Ontology enriched terms in the *MYCN* negative correlated genes in two neuroblastoma (NB) datasets (left panel: E-MTAB-1781, right panel: TARGET). Bar length represents NES absolute value while color intensity represents -log10 FDR. **(B)** Pathway enrichment for five select immune pathways (GO terms) in different cancer datasets. Symbol size and color intensity indicate—log10 FDR and NES. GO terms enriched in *MYCN* (left panel) and *MYC* (right panel) correlated genes. **(C)** Color intensity indicated the mean of T-helper subset relative abundance in *MYCN* amplified (MNA) and non-MNA patient expression profiles plotted as heatmap. **(D)** Macrophage relative abundance (left panel: M1 population, right panel: M2 population) in MNA and non-MNA patient gene expression profiles. Each symbol represents an individual patient (MNA = 122, non-MNA = 580), the middle line represents the median, the first and third quartiles are indicated as box limits, whiskers represents 1.5 box lengths, extreme values are indicated as single dots. Wilcoxon matched pair test; **P < 0.01; ****P < 0.0001.

In order to investigate which immune system pathways are associated with *MYCN* in NB, we performed GSEA analysis in NB patient datasets. Interestingly, *MYCN* negative correlated genes are significantly enriched of different immune system pathways in both NB cohort 1 (E-MTAB-1781) and NB cohort 2 (TARGET) (**Figure 1A**). Moreover, we performed differential expressed gene analysis, and found that non-MNA patients are enriched of immune pathways (**Figure S1A**). Furthermore, immune pathways represent a consistent part of the enriched pathways in the MNA patients and in the *MYCN* anti-correlated genes (**Figures S1B, C**). Collectively, these data suggest that *MYCN* is negatively associated with the immune system (especially associated to interferon gamma and phagocytosis) in MNA NB. Since, *MYCN* overexpression is present in a large group of tumors (4, 9, 44–48), we investigated if *MYCN* was also associated to immune suppression in different *MYCN*-expressing cancers (SCLC, RMS, RB, Wilms, AML, T-ALL). We observed that different pathways associated to Th1 are negatively correlated to *MYCN* in different cancer types (**Figures 1B**, **S2A, B**). Remarkably, despite *MYC* and *MYCN* are orthologs we did not find the same anti-correlation for *MYC* in these malignancies (**Figure 1B**). In this view, we investigated which T-helper subsets were enriched in NB. The results confirmed a significantly high abundance of Th1 in non-MNA patients, while Th2 and Th17 were enriched in MNA patients (**Figure 1C**). Furthermore, patients enriched for Th1 are not enriched for Th2/Th17 (**Figure S3**). As described before, Th1 cells are polarizing macrophages toward M1 phenotype, while Th2 direct macrophages polarization toward M2 (49). Thus, we investigated macrophage phenotype enrichment in NB, and found that M1 are significantly enriched in non-MNA patients while M2 are more abundant in MNA patients (**Figure 1D**).

### MYCN Exerts a Key Role in the Wide Neuroblastoma Immune Network

**Figure 2:**
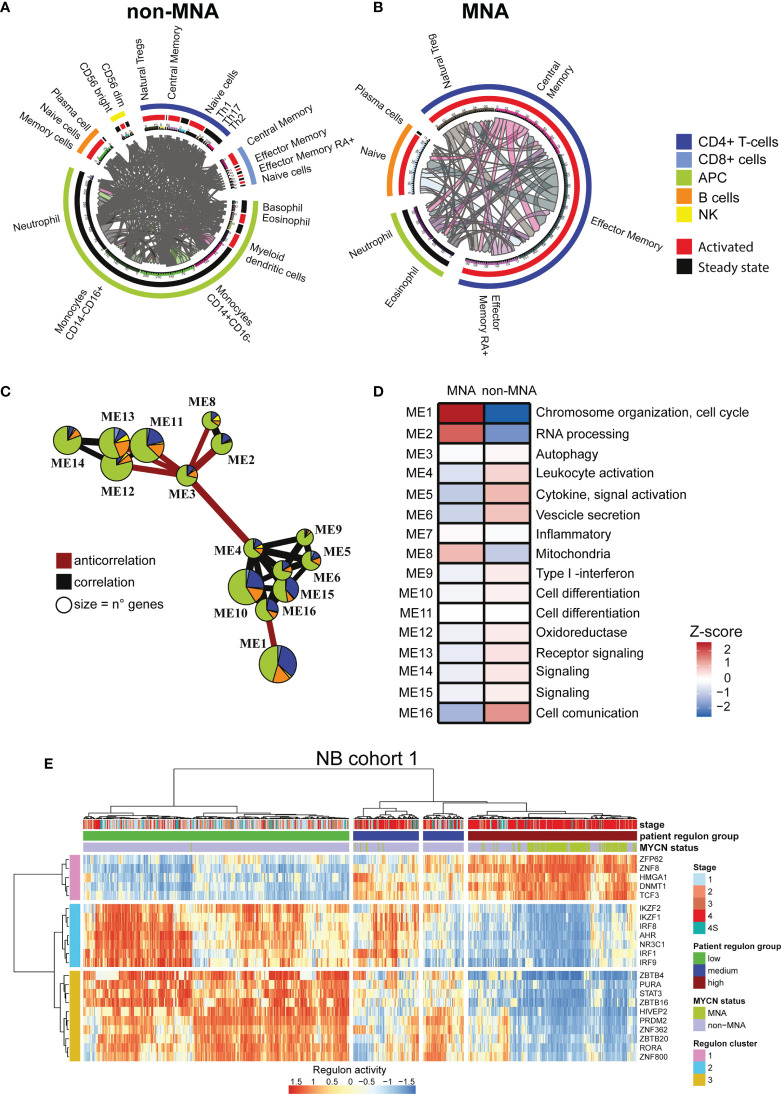
*MYCN* up-regulation impacts a wide immune interaction network. **(A, B)** Circular plots. Immune subpopulations are specified outside the circle, outer circle represents cell types, inner circle represents activation status. Connecting lines indicate connection between two subpopulations and are proportional to the number of connections. **(A)** Immune network in non-(*MYCN* amplification) MNA patients. **(B)** Immune network in MNA patients. **(C)** Immune system gene module network in neuroblastoma (NB). Edges size is proportional to Pearson correlation coefficients, correlation is indicated in gray and negative correlation in red. Modules with no connections are not shown, module size is proportional to the number of genes within. Pie chart colors correspond to immune cell types, the size of the slices corresponds to the number of the genes. **(D)** Heatmap of MNA and non-MNA patient gene expression profiles (MNA = 122, non-MNA = 580) in NB1 cohort (E-MTAB-1781). Color intensity is proportional to z-score of the average eigengenes for each gene module. Main pathway enrichment for each module is listed on the left, full list is present in the Supplementary Tables. **(E)** heatmap representing the normalize relative abundance of regulons in NB1 cohort (E-MTAB-1781). Hierarchical clustering is conducted on the row and the columns using the Euclidean distance. Clinical data are on top.

As *MYCN* overexpression deeply reprograms NB cells, we investigated the difference in immune network between MNA and non-MNA patients. We firstly identified differential expressed immune genes on the MNA and non-MNA which are present in the cell surface, and we mapped the protein-protein interaction between immune population. We found that non-MNA patients present a much more complex network than MNA patients and a more diverse population scenario (**Figures 2A, B**). Moreover, we identified differential expressed genes after *MYCN* silencing which sub-cellular locations is on the surface. Furthermore, we mapped their potential interactors on the immune population, showing that *MYCN* regulates a wide network of interactions in immune cells in the NB context (**Figures S4A, B**). We used an unbiased clustering approach to group genes in NB belonging to immune system with correlating expression patterns, and we annotated their functional properties through GO enrichment analysis. This analysis revealed 16 different modules that are differentially enriched in MNA and non-MNA patients (**Figures 2C–D**, and **S5A, B**). Interestingly, modules 1 and 2 that are enriched in MNA patients are functional annotated with chromosome organization, cell cycle, RNA processing. Modules containing immune activation genes are instead enriched in non-MNA (**Figure 2D**). Moreover, non-MNA are enriched in modules associated to extracellular vesicles, cytokine production and cell communication (**Figure 2D**). We also inferred the putative regulons in order to identify transcription factor dysregulated between MNA and non-MNA. We identified 22 regulons that are common between the two NB cohorts (**Figures 2E** and **S6A–D**), showing a similar enrichment pattern in MNA and non-MNA patients (**Figures 2E** and **S7A**) and interestingly these transcription factors are also connected through direct protein-protein interactions (**Figure S7B**). Therefore, we apply hierarchical clustering defining three regulon groups with transcriptional factors with similar activity (**Figures 2E** and **S7C**). These three-regulon clusters are differentially enriched of immune modules: regulon cluster one is enriched with genes related to cell-cycle while the other clusters group genes related to immunity (**Figures S7D, E**). Lastly, ChIP-seq public data analysis reveals that the regulon transcription factors are directly regulated by N-Myc (**Figures S8A, B**).

### MYCN Effect on Immune System Is an Independent Prognostic Indicator in Neuroblastoma

**Figure 3:**
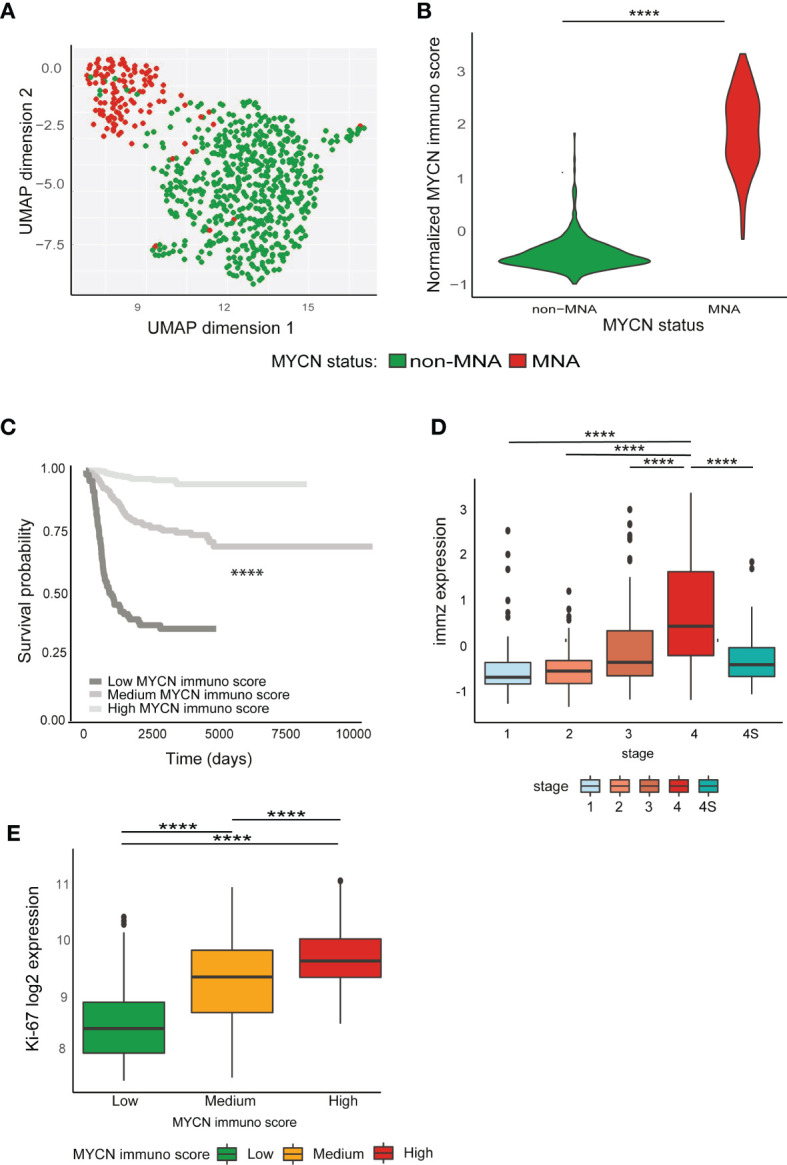
*MYCN* effect on immune system has a prognostic impact. **(A–E)** Analyses conducted on E-MTAB-1781 dataset. **(A)** Uniform Manifold Approximation and Projection (UMAP) projection of *MYCN* amplification (MNA) and non-MNA patient gene expression profiles (PGEP). **(B)** Violin plots represent normalized *MYCN* immune score in MNA and non-MNA PGEP. **(C)** Kaplan–Meier plots for the probability of overall survival over time for patients associated with *MYCN* immune score (high enriched, n = 114; medium enriched, n = 311; low enriched, n = 277). Associated P value is shown in the middle of the plot (log-rank test). **(D)** Normalized *MYCN* immune score in different International Neuroblastoma Staging System Committee (INSS) classification stages. **(E)** Ki-67 log2 expression in patients associated with *MYCN* immune. Wilcoxon matched pair test; ****P < 0.0001.

The identified regulon clusters showed a prognostic impact in both NB cohorts (**Figures S9A–D**). Therefore, we investigated the prognostic impact of *MYCN* regulation of the immune system using a logistic regression and penalized Cox regression, to identify which genes involved in the immune system are associated with the *MYCN* status and the prognosis (AUC = 0.97, **Figures S10A–C**). We built a *MYCN* immune score using the model weight to stratify the NB patients (**Figure 3A**). We identified 430 genes positively associated with MNA and 218 negatively associated. Moreover, we mined PubMed to check which genes in the signature were already identified in literature: 127 were already associated to NB and 60 to *MYCN* (**Figure S10D**). Interestingly, cluster 2-3 transcription factor (TF) regulons were found to negatively regulates *MYCN* positive associated immune signature and positively regulates the negatively associated genes (**Figure S10E**). The *MYCN* immune score was significantly enriched in MNA patients (**Figure 3B**) and according to the score we stratified the patients in three clusters (low, medium, and high *MYCN* immune dysregulation). Remarkably, the high *MYCN* immune dysregulation group was associated with poor prognosis while the low group with a favorable prognosis in NB (**Figure 3C**). Furthermore, the *MYCN* immune score was associated with stage 4 (**Figure 3D**) and high proliferation (**Figure 3E**). We confirmed in an additional NB cohort that the *MYCN* immune score was associated with *MYCN*-status, poor survival, stage, high proliferation and unfavorable histology (**Figures S11A–F**). We also confirmed with a different algorithm (50) that *MYCN* immune score is associated with low immune infiltration and high tumor purity (**Figures S12A, B**). Moreover, the *MYCN* immune score correlated with negative immune checkpoints and anti-correlated with positive immune checkpoints in both cohorts (**Figure S12C**). Interestingly, *MYCN* immune score correlated with Th2 cytokines while negatively associated with Th1 cytokines (**Figure S12D**). As aforementioned, MHC genes are poorly expressed in NB, we investigated whether *MYCN* immune score was associated to MHC genes. Indeed, we noticed that *MYCN* immune score anti-correlated with MHC genes (**Figure S12E**). We also found that *MYCN* immune score is also negatively associated to Toll Like Receptors, as a confirmation that immune receptors are negatively associated to *MYCN* (**Figure S12F**). Moreover, *MYCN* immune score was predictive of the survival in *MYCN* in non-MNA patients in both NB cohorts, while the *MYCN* expression did not (**Figures S13A–D**). Lastly, Cox multivariate analysis showed that *MYCN* immune score is an independent prognostic factor and significantly associated at overall and event free survival in both NB cohorts and also in non-MNA patients (**Figures S14A–H**).

### Anti-*MYCN* BGA002 Inhibits CD276 Expression and Restores Natural Killer Susceptibility in Neuroblastoma

**Figure 4:**
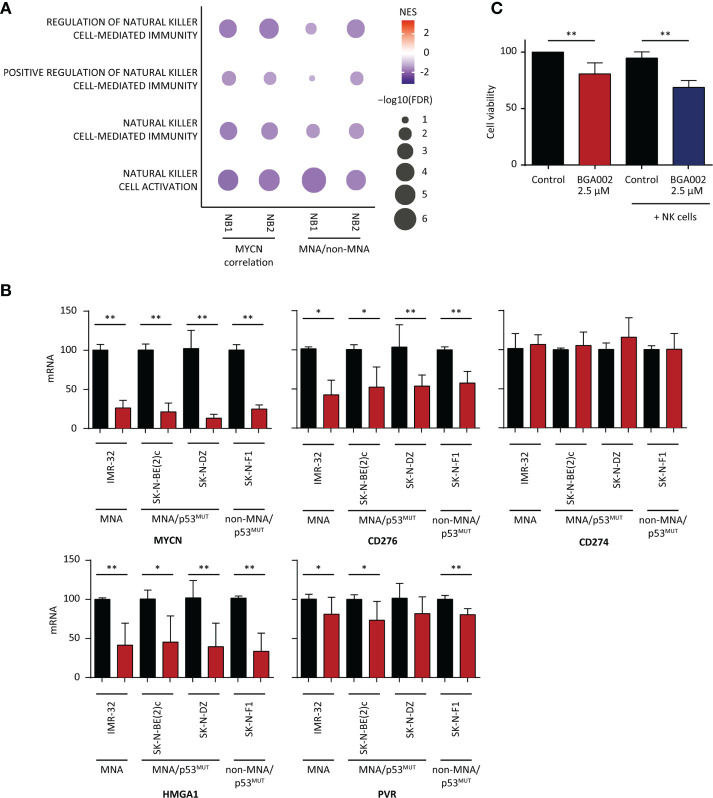
BGA002 blocks CD276 and restores neuroblastoma (NB) susceptibility to natural killer (NK) cells. **(A)** Pathway enrichment for four select immune pathways [Gene Ontology (GO) terms] associated to NK cells. (NB1: E-MTAB-1781, NB2: TARGET NB). Symbol size and color intensity indicate—log10 FDR and NES. GO terms enriched in *MYCN* (left) anti-correlated genes and GO terms enriched in non-*MYCN* amplification (MNA) (right) patients. **(B)** mRNA expression inhibition of different genes (MYCN, CD276, CD274, HMGA1, PVR) in NB cell lines measured through real-time PCR after 12 h of treatment with BGA002 2.5 µM (black is the control, red the treatment, n = 4 biological replicates for each cell line). Wilcoxon matched pair test; *P < 0.05, **P < 0.01, where not shown is not significant (P > 0.05). **(C)** Kelly-luc cell line (MNA NB cell line transfected with luciferase) viability after treatment with BGA002 2.5 µM and NK co-culture (five independent experiments). Wilcoxon matched pair test; **P < 0.01.

We found that NK related pathways are downregulated in MNA-NB patients in the two cohorts used in this study (**Figure 4A**). Indeed, we also found that MHC associated pathway are enriched in genes that are anti-correlating with MYCN (**Figure S15A**). MNA patient GEP showing a reduced expression of NK receptors (NKG2D and Nkp46) and a reduced expression of the cognate ligands (ULBP1, ULBP2, ULPB3, MICA, MICB) known as Self-induced antigen (**Figure S15A**). As reported in literature NK are dysregulated in NB and CD276 has been identified as one of the most relevant factors leading NK inhibition in NB (51–55). Moreover, we found CD276 expression higher in MNA *versus* non-MNA NB patients (**Figures S15B, C**). Thus, we investigated if *MYCN* blocking through the anti-*MYCN* antigene PNA oligonucleotide BGA002 could downregulates its expression in different NB cell lines (comprising MNA, p53 mutated, and non-MNA). Anti-*MYCN* BGA002 potently reduced *MYCN* expression and led to a significant CD276 down-regulation after the treatment in *MYCN*-expressing MNA and non-MNA NB cell lines (**Figure 4B**).

PD-L1 (also named CD274) expression has been reported in NB, but PD-L1 blockade immunotherapy has not reported to be effective in NB (56). We did not find association between neither the survival nor the *MYCN* immune score and PD-L1, while we found that its expression is higher in non-MNA NB patients in both NB cohorts (**Figure S15A**). Moreover, basal expression of CD274 was low (**Figure S15B**). In line with these finding, *MYCN* blocking by BGA002 did not lead to CD274 down-regulation (**Figure 4B**).

HMGA1 has been described as N-Myc transcriptional target (57) and linked to resistance to apoptosis, proliferation induction, and angiogenesis, while it is implicated in the mechanism of resistance to retinoic acid in NB (58–60). We found in the previous section that HMGA1 is a regulon in the cluster 1 associated with poor prognosis in both the NB cohorts, regulated different genes in the *MYCN* immune signature and highly expressed in MNA NB patients (**Figure S15A**). Therefore, we tested if *MYCN* inhibition by anti-*MYCN* BGA002 led to its down-regulation, and indeed we observed a dramatic HMGA1 reduction of expression (**Figure 4B**).

The analysis in the previous section showed that PVR is present in the *MYCN* immune score and negatively associated with the survival (**Supplementary Tables 10** and **11**) and its expression is higher in MNA patients (**Figure S15A**). However, the role of PVR in NB is debated, it has been reported to positively activate NK cells while it has been noticed the contrary in other malignancies where is also associated with poor outcome (61, 62). However, *MYCN* blocking by BGA002 led to PVR down-regulation in a small extent (**Figure 4B**).

Considering we found downregulated the NK pathways in MNA-NB patients in the two cohorts used in this study (**Figure 4A**) and because we also found CD276 down-regulation after *MYCN* inhibition by BGA002 (**Figure 4B**), we evaluated the potential effect of *MYCN* inhibition by BGA002 of the reactivation capacity on NK lysis of MNA NB cells (we used Kelly-luc, MNA cell-line transfected with luciferase). We did not notice viability decrease adding the NK-cells alone in co-culture with MNA-NB cells (**Figure 4C**). Indeed, we found that treatment with BGA002 in co-culture with NK in the MNA NB cells significantly impacted on cell viability (**Figure 4C**).

## Discussion

*MYCN* is known to influence diverse aspects of the cancer cells, dysregulating a large network of intracellular pathways (2). Despite previous indication that immune system in NB is altered, the role of *MYCN* in the immune response is not fully understood (19, 33). Since the high complexity showed by the immune system and the intricate relationship between itself and the cancer cells, proper system biology studies are required to map this complex network (63). Our results show that *MYCN* has a great role in dysregulating the immune network in NB, as we showed that different immune pathways are enriched in *MYCN* correlated genes and in MNA *versus* non-MNA differential expressed genes. Moreover, we confirmed in independent cancer mRNA expression patient cohorts that *MYCN* immune anti-correlation is not restricted to NB, but it is a feature also of other malignancies (small cell lung cancer, rhabdomyosarcoma, Wilms’ tumor, retinoblastoma, acute myeloid leukemia, and T-acute lymphoid leukemia) (4). Interestingly, we did not find the same anti-correlation pathway with *MYC*, suggesting a different behavior between the two oncogenes of the same family. We found that *MYCN* anti-correlated with Th1 immunity while correlated with Th2, and these subsets are mutually exclusive enriched in NB. As expected by that, *MYCN* correlated with M2 macrophages and inversely correlated with M1 subset. In line with previous literature, Th1 and M1 subsets are associated to anti-tumor immunity while Th2/Th17 and M2 are hijacked by the cancer cells to sustain their growth (64). Indeed, our results evidence a complex regulation in MNA *versus* non-MNA NB, where *MYCN* is a key player in remodeling the immunological micro-environment toward a suppressive phenotype.

We showed that mapping the possible protein-protein interactions between immune cell types in non-MNA NB patients revealed a complex immune network that is lost in MNA patients. Moreover, we found that *MYCN* expression regulated different genes involved in direct interactions with immune cell types playing as a driver of this poor immune environment. We identified 16 immune gene modules that are differently enriched in MNA and non-MNA NB patients, where modules related to immune receptors, signaling, and cytokines are enriched in the latter group. Therefore, all these results confirm a deeply dysregulated immune tumor micro-environment in NB. We also inferred the putative TFs that regulate the immune genes and differed between MNA and non-MNA NB, founding three regulon clusters (22 regulons in total) who are in common between NB1 and NB2 cohorts. Our results show that these regulon clusters are differentially enriched in MNA and non-MNA patients and they regulate the immune landscape in NB. We found that there are direct interactions between these TFs, as well as N-Myc also directly regulates the expression of these regulons as mechanistic explanation of the immune response dysregulation in NB.

Furthermore, we found a link between *MYCN* immune dysregulation and prognostic impact in NB. Generating a *MYCN* immune signature we stratified the NB cohorts in three groups (low, medium and high *MYCN* immune dysregulation) which showed a marked difference in the prognosis. Moreover, the *MYCN* immune score was also associated with other different NB characteristics as stage, proliferation, and histology and we confirmed these associations in an independent NB cohort. As a confirmation, the *MYCN* immune score correlated with immune checkpoints, Th cytokines, MHC genes, and TLRs capturing the immune landscape of the NB. Moreover, apart from MNA, there are other cancer events that can lead to dysregulated N-Myc higher activity (mRNA and protein stabilization, mi-RNA alteration, and so on) making difficult to infer the *MYCN* relevance in these cases (65, 66). Indeed, our score is able to capture this activity in non-MNA patients where *MYCN* mRNA expression level is not able to stratify the patients.

MNA and refractory NB patients are lacking viable therapeutic options (67, 68). Since the broad role of *MYCN* in the pathology, its restricted profile of expression during the embryonal stage, it is a promising target of intervention (69). Despite different attempts, it has been proven to be challenging to specifically target N-Myc with small molecules. We previously reported the specific *MYCN* inhibition through an anti-*MYCN* antigene oligonucleotide PNA (BGA002), showing *MYCN* inhibition and a therapeutic effect *in vitro* and *in vivo* (3, 10). Thus, we investigated if this specific inhibition exerted an effect on the immune suppression guided by *MYCN*. Our results showed that *MYCN* inhibition by BGA002 resulted in a cascade to downregulation of negative immune checkpoints (CD276) and regulons implied (HMGA1) in the immune-suppression phenotype. Indeed, we noticed that anti-*MYCN* treatment also led to NK lysis of MNA NB cells.

Collectively, the data here presented provide demonstrations of the broad role of *MYCN* in suppressing the immune landscape, which play a role in the poor prognosis associated to this oncogene. These data also suggest that *MYCN* blocking can ameliorate the immune suppression characterizing MNA NB patients. Indeed, while specific *MYCN* inhibition by anti-*MYCN* BGA002 can be proposed as a single treatment for MNA NB patients, our results also show that its activity can restore the responsiveness of the immune system against NB, opening the way to use anti-*MYCN* inhibition in combination with immune-therapy.

## Data Availability Statement

The datasets presented in this study can be found in online repositories. The names of the repository/repositories and accession number(s) can be found in the article/**Supplementary Material**.

## Ethics Statement

The studies involving human participants were reviewed and approved by the Scientific Ethical Committee of Bologna University. Written informed consent for participation was not required for this study in accordance with the national legislation and the institutional requirements.

## Author Contributions

SR performed the bioinformatic analysis. CA, SL, and LM performed the experiments and acquired and analyzed the biological data. MF provided dataset for the analysis. SR, DR, and RT wrote the manuscript. AP, PH, and MF revised the manuscript. AP, PH, MF, and RT acquired funds. SR and RT designed and supervised the study. All authors contributed to the article and approved the submitted version.

## Funding

RT, AP, and PH are funded by the University of Bologna. LM is funded by AGEOP. SR, CA, and SL are funded by BIOGENERA SpA; the funder was not involved in the study design, collection, analysis, interpretation of data, the writing of this article or the decision to submit it for publication.

## Conflict of Interest

RT and AP are co-founders and shareholders of Biogenera. Authors SR, SL and CA are employed by Biogenera.

The remaining authors declare that the research was conducted in the absence of any commercial or financial relationships that could be construed as a potential conflict of interest.

## References

[1] Mathsyaraja H Eisenman RN. Parsing Myc Paralogs in Oncogenesis. Cancer Cell (2016) 29:1–2. 10.1016/j.ccell.2015.12.009 26766585

[2] Ruiz-Pérez MV Henley AB Arsenian-Henriksson M. The MYCN Protein in Health and Disease. Genes (Basel) (2017) 8:113. 10.3390/genes8040113 PMC5406860 28358317

[3] Montemurro L Raieli S Angelucci S Bartolucci D Amadesi C Lampis S. A Novel MYCN-Specific Antigene Oligonucleotide Deregulates Mitochondria and Inhibits Tumor Growth in MYCN-Amplified Neuroblastoma. Cancer Res (2019) 79:6166–77. 10.1158/0008-5472.CAN-19-0008 31615807

[4] Rickman DS Schulte JH Eilers M. The Expanding World of N-MYC–Driven Tumors. Cancer Discov (2018) 8:150–63. 10.1158/2159-8290.CD-17-0273 29358508

[5] Huang M Weiss WA. Neuroblastoma and MYCN. Cold Spring Harb Perspect Med (2013) 3:22. 10.1101/cshperspect.a014415 PMC3784814 24086065

[6] Matthay KK Maris JM Schleiermacher G Nakagawara A Mackall CL Diller L. Neuroblastoma. Nat Rev Dis Primers (2016) 2:16078. 10.1038/nrdp.2016.78 27830764

[7] Zimmerman KA Yancopoulos GD Collum RG Smith RK Kohl NE Denis KA. Differential expression of myc family genes during murine development. Nature (1986) 319:780–3. 10.1038/319780a0 2419762

[8] Fletcher JI Ziegler DS Trahair TN Marshall GM Haber M Norris MD. Too many targets, not enough patients: rethinking neuroblastoma clinical trials. Nat Rev Cancer (2018) 18:389–400. 10.1038/s41568-018-0003-x 29632319

[9] Tonelli R McIntyre A Camerin C Walters ZS Leo KD Selfe J. Antitumor Activity of Sustained N-Myc Reduction in Rhabdomyosarcomas and Transcriptional Block by Antigene Therapy. Clin Cancer Res (2012) 18:796–807. 10.1158/1078-0432.CCR-11-1981 22065083

[10] Tonelli R Purgato S Camerin C Fronza R Bologna F Alboresi S. Anti-gene peptide nucleic acid specifically inhibits MYCN expression in human neuroblastoma cells leading to cell growth inhibition and apoptosis. Mol Cancer Ther (2005) 4:779–86. 10.1158/1535-7163.MCT-04-0213 15897242

[11] Campbell K Gastier-Foster JM Mann M Naranjo AH Van Ryn C Bagatell R. Association of MYCN copy number with clinical features, tumor biology, and outcomes in neuroblastoma: A report from the Children’s Oncology Group. Cancer (2017) 123:4224–35. 10.1002/cncr.30873 PMC5650521 28696504

[12] Seeger RC Brodeur GM Sather H Dalton A Siegel SE Wong KY. Association of multiple copies of the N-myc oncogene with rapid progression of neuroblastomas. N Engl J Med (1985) 313:1111–6. 10.1056/NEJM198510313131802 4047115

[13] Brodeur GM Seeger RC Schwab M Varmus HE Bishop JM. Amplification of N-myc in untreated human neuroblastomas correlates with advanced disease stage. Science (1984) 224:1121–4. 10.1126/science.6719137 6719137

[14] Brodeur GM. Neuroblastoma: biological insights into a clinical enigma. Nat Rev Cancer (2003) 3:203–16. 10.1038/nrc1014 12612655

[15] Nallasamy P Chava S Verma SS Mishra S Gorantla S Coulter DW. PD-L1, Inflammation, non-coding RNAs, and Neuroblastoma: Immuno-oncology Perspective. Semin Cancer Biol (2018) 52:53–65. 10.1016/j.semcancer.2017.11.009 29196189 PMC5972043

[16] Maris JM Matthay KK. Molecular biology of neuroblastoma. J Clin Oncol (1999) 17:2264–79. 10.1200/JCO.1999.17.7.2264 10561284

[17] Ward E DeSantis C Robbins A Kohler B Jemal A. Childhood and adolescent cancer statistics, 2014. CA Cancer J Clin (2014) 64:83–103. 10.3322/caac.21219 24488779

[18] Hanahan D Weinberg RA. Hallmarks of cancer: the next generation. Cell (2011) 144:646–74. 10.1016/j.cell.2011.02.013 21376230

[19] Mina M Boldrini R Citti A Romania P D’Alicandro V De Ioris M. Tumor-infiltrating T lymphocytes improve clinical outcome of therapy-resistant neuroblastoma. Oncoimmunology (2015) 4:e1019981. 10.1080/2162402X.2015.1019981 26405592 PMC4570119

[20] Shang B Liu Y Jiang S Liu Y. Prognostic value of tumor-infiltrating FoxP3+ regulatory T cells in cancers: a systematic review and meta-analysis. Sci Rep (2015) 5:15179. 10.1038/srep15179 26462617 PMC4604472

[21] Vanichapol T Chutipongtanate S Anurathapan U Hongeng S. Immune Escape Mechanisms and Future Prospects for Immunotherapy in Neuroblastoma. BioMed Res Int (2018) 2018:e1812535. 10.1155/2018/1812535 PMC5845499 29682521

[22] Challagundla KB Wise PM Neviani P Chava H Murtadha M Xu T. Exosome-mediated transfer of microRNAs within the tumor microenvironment and neuroblastoma resistance to chemotherapy. J Natl Cancer Inst (2015) 107. 10.1093/jnci/djv135 PMC4651042 25972604

[23] Russell HV Hicks J Okcu MF Nuchtern JG. CXCR4 expression in neuroblastoma primary tumors is associated with clinical presentation of bone and bone marrow metastases. J Pediatr Surg (2004) 39:1506–11. 10.1016/j.jpedsurg.2004.06.019 15486895

[24] Majzner RG Simon JS Grosso JF Martinez D Pawel BR Santi M. Assessment of programmed death-ligand 1 expression and tumor-associated immune cells in pediatric cancer tissues. Cancer (2017) 123:3807–15. 10.1002/cncr.30724 28608950

[25] Bach J-P Rinn B Meyer B Dodel R Bacher M. Role of MIF in inflammation and tumorigenesis. Oncology (2008) 75:127–33. 10.1159/000155223 18791328

[26] Merchant MS Wright M Baird K Wexler LH Rodriguez-Galindo C Bernstein D. Phase I Clinical Trial of Ipilimumab in Pediatric Patients with Advanced Solid Tumors. Clin Cancer Res (2016) 22:1364–70. 10.1158/1078-0432.CCR-15-0491 PMC5027962 26534966

[27] Burr ML Sparbier CE Chan KL Chan Y-C Kersbergen A Lam EYN. An Evolutionarily Conserved Function of Polycomb Silences the MHC Class I Antigen Presentation Pathway and Enables Immune Evasion in Cancer. Cancer Cell (2019) 36:385–401.e8. 10.1016/j.ccell.2019.08.008 31564637 PMC6876280

[28] Richards RM Sotillo E Majzner RG. CAR T Cell Therapy for Neuroblastoma. Front Immunol (2018) 9:2380. 10.3389/fimmu.2018.02380 30459759 PMC6232778

[29] Bernards R Dessain SK Weinberg RA. N-myc amplification causes down-modulation of MHC class I antigen expression in neuroblastoma. Cell (1986) 47:667–74. 10.1016/0092-8674(86)90509-x 3096575

[30] Layer JP Kronmüller MT Quast T van den Boorn-Konijnenberg D Effern M Hinze D. Amplification of N-Myc is associated with a T-cell-poor microenvironment in metastatic neuroblastoma restraining interferon pathway activity and chemokine expression. OncoImmunology (2017) 6:e1320626. 10.1080/2162402X.2017.1320626 28680756 PMC5486176

[31] Brandetti E Veneziani I Melaiu O Pezzolo A Castellano A Boldrini R. MYCN is an immunosuppressive oncogene dampening the expression of ligands for NK-cell-activating receptors in human high-risk neuroblastoma. Oncoimmunology (2017) 6:e1316439. 10.1080/2162402X.2017.1316439 28680748 PMC5486189

[32] Melaiu O Mina M Chierici M Boldrini R Jurman G Romania P. PD-L1 Is a Therapeutic Target of the Bromodomain Inhibitor JQ1 and, Combined with HLA Class I, a Promising Prognostic Biomarker in Neuroblastoma. Clin Cancer Res (2017) 23:4462–72. 10.1158/1078-0432.CCR-16-2601 28270499

[33] Borriello L Seeger RC Asgharzadeh S DeClerck YA. More than the genes, the tumor microenvironment in neuroblastoma. Cancer Lett (2016) 380:304–14. 10.1016/j.canlet.2015.11.017 PMC5558454 26597947

[34] George J Lim JS Jang SJ Cun Y Ozretić L Kong G. Comprehensive genomic profiles of small cell lung cancer. Nature (2015) 524:47–53. 10.1038/nature14664 26168399 PMC4861069

[35] Subramanian A Tamayo P Mootha VK Mukherjee S Ebert BL Gillette MA. A knowledge-based approach for interpreting genome-wide expression profiles. PNAS (2005) 102:15545–50. 10.1073/pnas.0506580102 PMC1239896 16199517

[36] Newman AM Steen CB Liu CL Gentles AJ Chaudhuri AA Scherer F. Determining cell type abundance and expression from bulk tissues with digital cytometry. Nat Biotechnol (2019) 37:773–82. 10.1038/s41587-019-0114-2 PMC6610714 31061481

[37] Thul PJ Åkesson L Wiking M Mahdessian D Geladaki A Ait Blal H. A subcellular map of the human proteome. Science (2017) 356(6340):eaal3321. 10.1126/science.aal3321 28495876

[38] Rieckmann JC Geiger R Hornburg D Wolf T Kveler K Jarrossay D. Social network architecture of human immune cells unveiled by quantitative proteomics. Nat Immunol (2017) 18:583–93. 10.1038/ni.3693 28263321

[39] Feng C Song C Liu Y Qian F Gao Y Ning Z. KnockTF: a comprehensive human gene expression profile database with knockdown/knockout of transcription factors. Nucleic Acids Res (2020) 48:D93–D100. 10.1093/nar/gkz881 31598675 PMC6943067

[40] Zhang B Horvath S. A general framework for weighted gene co-expression network analysis. Stat Appl Genet Mol Biol (2005) 4(1):17. 10.2202/1544-6115.1128. Article17. 16646834

[41] Langfelder P Horvath S. WGCNA: an R package for weighted correlation network analysis. BMC Bioinformatics (2008) 9:559. 10.1186/1471-2105-9-559 19114008 PMC2631488

[42] Goeman JJ. L1 penalized estimation in the Cox proportional hazards model. Biom J (2010) 52:70–84. 10.1002/bimj.200900028 19937997

[43] Becht E McInnes L Healy J Dutertre C-A Kwok IWH Ng LG. Dimensionality reduction for visualizing single-cell data using UMAP. Nat Biotechnol (2019) 37:38–44. 10.1038/nbt.4314 30531897

[44] Brägelmann J Böhm S Guthrie MR Mollaoglu G Oliver TG Sos ML. Family matters: How MYC family oncogenes impact small cell lung cancer. Cell Cycle (2017) 16:1489–98. 10.1080/15384101.2017.1339849 PMC5584863 28737478

[45] Lee WH Murphree AL Benedict WF. Expression and amplification of the N-myc gene in primary retinoblastoma. Nature (1984) 309:458–60. 10.1038/309458a0 6728001

[46] Hirvonen H Hukkanen V Salmi TT Pelliniemi TT Alitalo R. L-myc and N-myc in hematopoietic malignancies. Leuk Lymphoma (1993) 11:197–205. 10.3109/10428199309086996 8260894

[47] van Lohuizen M Breuer M Berns A. N-myc is frequently activated by proviral insertion in MuLV-induced T cell lymphomas. EMBO J (1989) 8:133–6. 10.1002/j.1460-2075.1989.tb03357.x PMC400781 2653809

[48] Williams RD Al-Saadi R Chagtai T Popov S Messahel B Sebire N. Subtype-specific FBXW7 mutation and MYCN copy number gain in Wilms’ tumor. Clin Cancer Res (2010) 16:2036–45. 10.1158/1078-0432.CCR-09-2890 PMC5122447 20332316

[49] Orecchioni M Ghosheh Y Pramod AB Ley K. Macrophage Polarization: Different Gene Signatures in M1(LPS+) vs. Classically and M2(LPS-) vs. Alternatively Activated Macrophages. Front Immunol (2019) 10:1084. 10.3389/fimmu.2019.01084 31178859 PMC6543837

[50] Yoshihara K Shahmoradgoli M Martínez E Vegesna R Kim H Torres-Garcia W. Inferring tumour purity and stromal and immune cell admixture from expression data. Nat Commun (2013) 4:2612. 10.1038/ncomms3612 24113773 PMC3826632

[51] Castellanos JR Purvis IJ Labak CM Guda MR Tsung AJ Velpula KK. B7-H3 role in the immune landscape of cancer. Am J Clin Exp Immunol (2017) 6:66–75. 28695059 PMC5498853

[52] Castriconi R Dondero A Augugliaro R Cantoni C Carnemolla B Sementa AR. Identification of 4Ig-B7-H3 as a neuroblastoma-associated molecule that exerts a protective role from an NK cell-mediated lysis. Proc Natl Acad Sci USA (2004) 101:12640–5. 10.1073/pnas.0405025101 PMC515110 15314238

[53] Khan M Arooj S Wang H. NK Cell-Based Immune Checkpoint Inhibition. Front Immunol (2020) 11:167. 10.3389/fimmu.2020.00167 32117298 PMC7031489

[54] Lee Y-H Martin-Orozco N Zheng P Li J Zhang P Tan H. Inhibition of the B7-H3 immune checkpoint limits tumor growth by enhancing cytotoxic lymphocyte function. Cell Res (2017) 27:1034–45. 10.1038/cr.2017.90 PMC5539354 28685773

[55] Pistoia V Morandi F Bianchi G Pezzolo A Prigione I Raffaghello L. Immunosuppressive microenvironment in neuroblastoma. Front Oncol (2013) 3:167. 10.3389/fonc.2013.00167 23805414 PMC3693127

[56] Jabbari P Hanaei S Rezaei N. State of the art in immunotherapy of neuroblastoma. Immunotherapy (2019) 11:831–50. 10.2217/imt-2019-0018 31094257

[57] Giannini G Cerignoli F Mellone M Massimi I Ambrosi C Rinaldi C. High mobility group A1 is a molecular target for MYCN in human neuroblastoma. Cancer Res (2005) 65:8308–16. 10.1158/0008-5472.CAN-05-0607 16166307

[58] Petroni M Veschi V Gulino A Giannini G. Molecular mechanisms of MYCN-dependent apoptosis and the MDM2-p53 pathway: an Achille’s heel to be exploited for the therapy of MYCN-amplified neuroblastoma. Front Oncol (2012) 2:141. 10.3389/fonc.2012.00141 23091802 PMC3470040

[59] Zaatiti H Abdallah J Nasr Z Khazen G Sandler A Abou-Antoun TJ. Tumorigenic proteins upregulated in the MYCN-amplified IMR-32 human neuroblastoma cells promote proliferation and migration. Int J Oncol (2018) 52:787–803. 10.3892/ijo.2018.4236 29328367 PMC5807036

[60] Dobrotkova V Chlapek P Jezova M Adamkova K Mazanek P Sterba J. Prediction of neuroblastoma cell response to treatment with natural or synthetic retinoids using selected protein biomarkers. PloS One (2019) 14:e0218269. 10.1371/journal.pone.0218269 31188873 PMC6561640

[61] Stamm H Klingler F Grossjohann E-M Muschhammer J Vettorazzi E Heuser M. Immune checkpoints PVR and PVRL2 are prognostic markers in AML and their blockade represents a new therapeutic option. Oncogene (2018) 37:5269–80. 10.1038/s41388-018-0288-y PMC6160395 29855615

[62] Zhou X Du J Wang H Chen C Jiao L Cheng X. Repositioning liothyronine for cancer immunotherapy by blocking the interaction of immune checkpoint TIGIT/PVR. Cell Commun Signal (2020) 18:142. 10.1186/s12964-020-00638-2 32894141 PMC7487564

[63] Yang Y. Cancer immunotherapy: harnessing the immune system to battle cancer. J Clin Invest (2015) 125:3335–7. 10.1172/JCI83871 PMC4588312 26325031

[64] Tay RE Richardson EK Toh HC. Revisiting the role of CD4 + T cells in cancer immunotherapy—new insights into old paradigms. Cancer Gene Ther (2020), 1–13. 10.1038/s41417-020-0183-x PMC7886651 32457487

[65] Otto T Horn S Brockmann M Eilers U Schüttrumpf L Popov N. Stabilization of N-Myc Is a Critical Function of Aurora A in Human Neuroblastoma. Cancer Cell (2009) 15:67–78. 10.1016/j.ccr.2008.12.005 19111882

[66] Powers JT Tsanov KM Pearson DS Roels F Spina CS Ebright R. Multiple mechanisms disrupt the let-7 microRNA family in neuroblastoma. Nature (2016) 535:246–51. 10.1038/nature18632 PMC4947006 27383785

[67] Mallepalli S Gupta MK Vadde R. Neuroblastoma: An Updated Review on Biology and Treatment. Curr Drug Metab (2019) 20:1014–22. 10.2174/1389200221666191226102231 31878853

[68] Pastor ER Mousa SA. Current management of neuroblastoma and future direction. Crit Rev Oncol Hematol (2019) 138:38–43. 10.1016/j.critrevonc.2019.03.013 31092383

[69] Dikic I Elazar Z. Mechanism and medical implications of mammalian autophagy. Nat Rev Mol Cell Biol (2018) 19:349–64. 10.1038/s41580-018-0003-4 29618831

