# Supplementary material for: MYCN Drives a Tumor Immunosuppressive Environment Which Impacts Survival in Neuroblastoma
Source: Front Oncol. 2021 Feb 25;11:625207. doi: 10.3389/fonc.2021.625207 (PMC7951059; doi:10.3389/fonc.2021.625207)
Supplement: Supplementary file 2 [file DataSheet_2.pdf]

Supplementary Methods

Transcriptional regulatory networks

Neuroblastoma expression datasets (E-MTAB-1781, TARGET dataset) were pre-processed as described before. We filter the cohorts for the genes selected by the WGCNA analysis (we considered only the genes present in the 16 modules, genes not assigned to any module (module 0) where discarded. The list of transcriptional factors were obtained from Lambert et all (1).We used the RTN package (2–4) to reconstruct the transcriptional network. We selected a p-value cut-off (1 *10e-5). We also selected eps = 0 (no tolerance) for the ARACNe algorithm (5). All the other standard options were maintained. We conducted the enrichment for the retrieved regulons in the differential expressed genes between MYCN amplified patients versus no-MNA (we obtained the list as described before). We considered the top 50 regulons (absolute dES) and we selected the regulons in common between NB1 (E-MTAB-1781) and NB2 (TARGET neuroblastoma) cohorts. List of the top 50 regulons is present in the supplementary tables (NB1 top 50 regulon in supplementary table 6, NB2 top 50 regulon in supplementary table 7). Correlation between regulons was conducted using Pearson correlation followed by hierarchical clustering on the obtained matrix. Jaccard coefficient were calculated on the selected regulons in the NB1 and NB2. Protein Protein interaction were downloaded from STRING database together with the binding annotation, we selected only the interaction with a combined score higher than 400 (6). The regulons where divided in three cluster using hierarchical clustering (ward method and correlation as a distance). The regulated genes in each regulons where used for pathway enrichment using GSEA software (list of the top 20 positively and negatively enriched pathways for each regulon cluster is present in Supplementary Table 8). We also calculated enrichment for each regulon cluster in the previous identifies genes modules, and we verify the enrichment for different signature in the regulons (considering the positive regulated genes for each regulon). List of the N-Myc chip was downloaded from AnimalTFDB (7), the MYCN Chip Score was calculated as the average of the score. The top N-Myc enriched regulons were used to build the transcriptional network with the RedeR package.

Patient expression profile clustering

The patient expression profiles clustering from NB1 cohort were clustered with hierarchical clustering (ward method and correlation as a distance). The four obtained cluster were grouped in three clusters and we calculate Kaplan-Meyer survival curve. The three clusters were defined as follow: low risk group was defined as highly active in regulon cluster 2 and 3, high risk group as poorly active in in regulon cluster 2 and 3 and highly active in regulon cluster 1, medium group showing intermediate features.

Autoencoder for dimension reduction

We used autoencoder for dimension reduction to obtain a 2d projection. We use keras library for rstudio. As input for the neural network we used the regulon activity for the transcription factors present in the three

clusters. We use a (12x6x2x6x12 neuron network) with dropout and leaky ReLU as activating function. We used Adam optimizer (learning rate = 0.001, weight decay = 0.0001) and mean squared error as loss function.

PubMed text mining

We download neuroblastoma and MYCN abstracts querying PubMed. We filtered out review articles and we selected only articles in English. The neuroblastoma query was restricted to the last ten years (both queries were conducted in July 2020). We downloaded all the abstract, we cleaned for stop words and punctation, and the abstract (and titles) were tokenized. We then checked which genes of the *MYCN* immune signature were present in the neuroblastoma and *MYCN* abstracts, and the results summarized as Venn diagrams. Genes found in the neuroblastoma and MYCN queries are listed in supplementary table 11.

Tumour purity estimation

Neuroblastoma expression datasets (E-MTAB-1781, TARGET dataset) were used as file input for the ESTIMATE algorithm in R (8).

Cox multivariate regression analysis

Neuroblastoma expression datasets (E-MTAB-1781, TARGET dataset) were used as input for the analysis. The cohorts were processed as described before. Clinical data were retrieved from the meta-data associated to expression files. MYCN immune score was defined as described before. Sex and MYCN amplification status were selected as covariates. The model was fitted using survival package in R.

Statistical analysis and software

Analysis were conducted in R (Rstudio) and Python (anaconda release). The following libraries from Python (version 3.7) were used: Scikit-learn, Matplotlib, matplotlib.pyplot, Pandas, UMAP, numpy. The following libraries from R (version 3.5) were used for analysis and graphs : ggplot2, dplyr, data.table, tydr, survival, survminer, wordcloud, WGCNA, circlize, iGraph, anRichment, stringr, ESTIMATE, Pheatmap, RTN, corrplot, RedeR, VennDiagram, tidytext, rentrez, RISmed, XML.

Supplementary bibliography

1. Lambert SA, Jolma A, Campitelli LF, Das PK, Yin Y, Albu M, Chen X, Taipale J, Hughes TR, Weirauch MT. The Human Transcription Factors. *Cell* (2018) **172**:650–665. doi:10.1016/j.cell.2018.01.029

2. Campbell TM, Castro MAA, de Santiago I, Fletcher MNC, Halim S, Prathalingam R, Ponder BAJ, Meyer KB. FGFR2 risk SNPs confer breast cancer risk by augmenting oestrogen responsiveness. *Carcinogenesis* (2016) **37**:741–750. doi:10.1093/carcin/bgw065

3. Castro MAA, de Santiago I, Campbell TM, Vaughn C, Hickey TE, Ross E, Tilley WD, Markowetz F, Ponder BAJ, Meyer KB. Regulators of genetic risk of breast cancer identified by integrative network analysis. *Nature Genetics* (2016) **48**:12–21. doi:10.1038/ng.3458

4. Fletcher MNC, Castro MAA, Wang X, de Santiago I, O'Reilly M, Chin S-F, Rueda OM, Caldas C, Ponder BAJ, Markowetz F, et al. Master regulators of FGFR2 signalling and breast cancer risk. *Nature Communications* (2013) **4**:2464. doi:10.1038/ncomms3464

5. Margolin AA, Nemenman I, Basso K, Wiggins C, Stolovitzky G, Favera RD, Califano A. ARACNE: An Algorithm for the Reconstruction of Gene Regulatory Networks in a Mammalian Cellular Context. *BMC Bioinformatics* (2006) **7**:S7. doi:10.1186/1471-2105-7-S1-S7

6. Szklarczyk D, Gable AL, Lyon D, Junge A, Wyder S, Huerta-Cepas J, Simonovic M, Doncheva NT, Morris JH, Bork P, et al. STRING v11: protein-protein association networks with increased coverage, supporting functional discovery in genome-wide experimental datasets. *Nucleic Acids Res* (2019) **47**:D607–D613. doi:10.1093/nar/gky1131

7. AnimalTFDB 3.0: a comprehensive resource for annotation and prediction of animal transcription factors | Nucleic Acids Research | Oxford Academic. Available at: https://academic.oup.com/nar/article/47/D1/D33/5094755?guestAccessKey=d0b5ab2d-e4ea-4b97-a181-51b578f1fa83 [Accessed June 16, 2020]

8. Yoshihara K, Shahmoradgoli M, Martínez E, Vegesna R, Kim H, Torres-Garcia W, Treviño V, Shen H, Laird PW, Levine DA, et al. Inferring tumour purity and stromal and immune cell admixture from expression data. *Nature Communications* (2013) **4**:2612. doi:10.1038/ncomms3612

Supplementary Figure legends

Supplementary Figure 1. MYCN is associated with immune pathway repression in neuroblastoma. **(A)** Bar plot represents Gene Ontology enriched term in the differential expressed genes (between *MYCN* amplified (MNA) and non-MNA gene expression profiles) in two neuroblastoma datasets (left panel: E-MTAB-1781, right panel: TARGET). Bar length represents NES absolute value while colour intensity represents -Log10 FDR. **(B)** Pie chart represent percentage of immune related pathways of the first 100 enriched pathways. First line: E-MTAB-1781, second line: TARGET. From left to right, 100 first pathways enriched in MNA and non-MNA differential expressed genes, pathway enriched in correlated and anti-correlated genes with MYCN (**C)** Number of pathways in common. Left panel: comparison between pathways enriched in E-MTAB-1781 MNA gene expression profiles, TARGET MNA gene expression profiles, E-MTAB-1781 *MYCN* positive correlated genes and TARGET *MYCN* positive correlated genes. Right panel: comparison between pathways enriched in E-MTAB-1781 non-MNA gene expression profiles, TARGET non-MNA gene expression profiles, E-MTAB-1781 *MYCN* negative correlated genes and TARGET *MYCN* negative correlated genes.

Supplementary Figure 2. MYCN is associated with immune pathway repression in different cancer datasets. **(A)** Table showing number of immune related pathways of the first 100 enriched pathways (enriched in the positive correlated or in the negative correlated genes. **(B)** Example of GSEA plot for GO ADAPTIVE IMMUNE RESPONSE for E-MTAB-1781 MYCN negative correlated genes, TARGET *MYCN* negative correlated genes, E-MTAB-1781 non-MNA gene expression profiles and TARGET non-MNA gene expression profiles.

Supplementary Figure 3. Exclusive presence of TH1/TH2 subsets in neuroblastoma. **(A)** heatmap representing the normalize relative abundance of TH subsets in NB1 cohort (E-MTAB-1781). Hierarchical clustering is conducted on the row and the columns using the Euclidean distance. Colour intensity represents z-score relative abundance of the Th subset.

Supplementary Figure 4. MYCN up-regulation impacts a wide immune interaction network. (**A-B)** Subpopulations are specified outside the circle, outer circle represents cell types, inner circle represents activation status. Connecting lines indicate connection between NB and immune subpopulation and are proportional to the number of connections. (**A)** *MYCN* negative regulated genes and the immune partners. (**B)** *MYCN* positive regulated genes and the immune partners.

Supplementary Figure 5. Correlation between immune WGCNA modules. **(A)** heatmap representing Pearson correlation between the module eigengenes (**B)** heatmap representing immune gene modules in neuroblastoma (on the vertical axis) and immune cell population on the horizontal axis. Colour intensity is proportional to z-score of the number of expressed proteins associated to each population.

Supplementary Figure 6. A conserved regulon pattern in neuroblastoma. **(A-D)** Correlation and overlap between the regulons (common regulons identified in NB1 (E-MTAB-1781) and NB2 (TARGET neuroblastoma)

cohorts) **(A)** Correlation between the regulons in NB1, boxes are identified by hierarchical clustering (**B)** Overlap between the regulons in NB1 using Jaccard coefficient. **(C)** Correlation between the regulons in NB2, boxes are identified by hierarchical clustering (**D)** Overlap between the regulons in NB2 using Jaccard coefficient.

Supplementary Figure 7. MNA different transcriptional regulons clusters have different immune features. **(A)** Differentially regulated regulons in MNA versus non-MNA patients (common regulons identified in NB1 (E-MTAB-1781) and NB2 (TARGET neuroblastoma) cohorts). Right panel: Bars are proportional to dES enrichment in NB1 cohorts. Colour shade indicate the number of elements present in the regulons. Left panel: Bars are proportional to dES enrichment in NB2 cohorts. Colour shade indicate the number of elements present in the regulons. **(B)** Protein-protein interaction between the transcription factors. The different colours indicate different type of interactions, grey colour indicate un-specified protein-protein interaction. **(C)** Hierarchical cluster dendrogram of the different transcriptional regulons. Hierarchical clustering is conducted on regulon activity in the NB1 patient cohort using the Euclidean distance. **(D)** Each different bar plot is showing enrichment for a different signature in each of the regulon clusters (regulon cluster 1 bar is not indicated in the last two bar plot as number of genes is zero). Bar plots showing number of genes positively regulated by the different cluster transcription factors. **(E)** Immune system gene module network in NB. Edges size is proportional to Pearson correlation coefficients, correlation is indicated in black and negative correlation in red. Modules with no connections are not shown, module size is proportional to the number of genes within. Pie chart colours correspond to positively and negatively regulated genes by the transcription factors present in each regulon cluster (one network for each regulon cluster), the size of the slices corresponds to the number of the genes.

Supplementary Figure 8. MYCN directly regulates transcription factors present in the regulator network **(A)** MYCN ChIP enrichment score in the transcription factors. **(B)** Transcription network of the top regulated N-Myc ChIP enriched. Square nodes indicate transcription factors and round nodes indicate inferred targets.

Supplementary Figure 9. Regulon clusters differentially impact the survival **(A)** regulon cluster projection obtained by autoencoder dimensionally reduction of the regulon activity. Colours indicate the regulon clusters, each dot represents a patient. **(B)** Kaplan–Meier plots for the probability of overall survival over time for patients in the NB1 cohort associated with regulon cluster (low risk, n = 345; medium risk, n = 138, high risk, n = 219). Associated P value is shown in the middle of the plot (log-rank test). (**C)** heatmap representing the normalize relative abundance of regulons in NB2 cohort. Hierarchical clustering is conducted on the row and the columns using the Euclidean distance. Clinical data are on top. **(D)** Kaplan–Meier plots for the probability of overall survival over time for patients in the NB2 cohort associated with regulon cluster (low risk, n =121; medium risk, n =33, high risk, n =89). Associated P value is shown in the middle of the plot (log-rank test).

Supplementary Figure 10. *MYCN* effect on immune system has a prognostic impact. (**A-B)** Wordclouds represent genes present in the *MYCN* immune score, ranked by normalized weight, the size is proportional to the associated weight. (**A)** Positive weight genes **(B)** Negative weight genes. (**C)** example of a Receiver Operating Characteristic (ROC) curve for logistic regression model (1 of 10 calculated). **(D)** Venn diagrams showing number MYCN signature genes found in PubMed text mining (genes present in neuroblastoma and MYCN related abstracts). Left panel: MYCN positive associated genes in the signature, right panel: MYCN negative associated genes in the signature. **(E)** left panel: Sankey plot of the transcription factor (TF) in the Regulon clusters and the regulated genes in the MYCN immune signature. Regulon cluster TF are order according to the identified cluster, the genes are order according their signature status (MYCN positive and negative associated). The colours of the line identify positive or negative regulations. Right panel: the Sankey plot is equivalent to the left panel, the interactions are grouped and summarized by regulon clusters.

Supplementary Figure 11. *MYCN* effect on immune system has a prognostic impact. **(A-F)** Analyses conducted on TARGET dataset (NB 2 cohort). **(A)** normalized *MYCN* immune score in MNA and non-MNA PGEP. (**B)** Kaplan–Meier plots for the probability of overall survival over time for patients associated with *MYCN* immune score (low enriched, n = 102; low enriched, n = 91; high enriched, n = 50). Associated P value is shown in the middle of the plot (log-rank test). **(C)** Kaplan–Meier plots for the probability of overall survival over time for patients associated with *MYCN* amplification status (MNA, n = 68; non-MNA, n = 175). Associated P value is shown in the middle of the plot (log-rank test). **(D)** normalized *MYCN* immune score in different INSS classification stages. (**E)** Ki-67 Log2 expression in patients associated with *MYCN* immune score. (**F)** normalized *MYCN* immune score associated with the histology. Wilcoxon matched pair test; * P < 0.05, ** P < 0.01, ****, P < 0.0001.

Supplementary Figure 12. MYCN immune score correlation with immune system components. **(A-B)** ESTIMATE enrichment for immunoScore, stromalScore, EstimateScore and TumorPurity in NB1 **(A)** and NB2 **(B)** cohort. **(C-F)** Colour bar represent Spearman correlation between MYCN immune score and different immune genes in E-MTAB-1781 and TARGET datasets. **(A)** Toll Like Receptor genes. (**B)** Positive (above) and negative (below) immune checkpoints. (**C)** T-helper cytokines. **(D)** MHC I and II genes.

Supplementary Figure 13. MYCN immune score correlation with immune system components. **(A-D)** Kaplan–Meier plots for the probability of overall survival over time for non-MNA patients. Associated P value is shown in the middle of the plot (log-rank test n.s. not significant, ** P < 0.01, ***, P < 0.001.). **(A)** E-MTAB1781 patient stratified for high and low MYCN immune score (low enriched, n = 100; medium enriched, n = 68; high enriched, n = 7). (**B)** E-MTAB1781 patient stratified for high and low MYCN expression (z-score higher than 1, high MYCN, n = 480, low MYCN, n = 532). (**C)** TARGET patient stratified for high and low MYCN immune score (low enriched, n = 100; medium enriched, n = 68; high enriched, n = 7). (**D)** TARGET patient stratified for high and low MYCN expression (z-score higher than 1, high MYCN, n = 25, low MYCN, n = 150).

Supplementary Figure 14. Immune score has a prognostic impact. **(A-H)** Cox multivariate regression analysis on NB1 (E-MTAB-1781) and NB2 (TARGET neuroblastoma) cohorts. Box plot that are on the left of the central line are positively associated with the hazard, Box plot that are on the right of the central line are negatively associated with the hazard. **(E-H)** Cox multivariate regression analysis on MYCN non-amplified NB1 (E-MTAB-1781) and NB2 (TARGET neuroblastoma) cohorts.

Supplementary Figure 15. Immune checkpoint expression in neuroblastoma. **(A)** Pathway enrichment for four select immune pathways (GO terms) associated to MHC and antigen processing. (NB1: E-MTAB-1781, NB2: TARGET NB). Symbol size and colour intensity indicate – log10 FDR and NES. GO terms enriched in *MYCN* (left) anti-correlated genes and GO terms enriched in non-MNA (right) patients. (**B)** heatmap of MNA and non-MNA patient gene expression profiles in NB1 cohort (E-MTAB-1781, MNA = 122, non-MNA = 580) and NB2 cohort (TARGET, MNA = 68, non-MNA = 175). Colour intensity is proportional to z-score of the average gene for each group. MICA gene is not present in the NB2 cohort and for this reason not indicated. **(C)** density plot representing mRNA expression of different immune checkpoint in neuroblastoma divided for MNA and non-MNA patients: on the left, NB1 cohort (E-MTAB1781) and on the right NB2 cohort (TARGET). The line in the middle is representing the median. From the top to the bottom: CD274, CD276, PVR, CTLA4. **(D)** mRNA basal expression of different genes (from left to right: CD276, CD274, HMGA1, PVR) in NB cell lines measured through Real-Time PCR after 12 hours of treatment with BGA002 2.5 µM (black is the control, red the treatment, n = 3 biological replicates for each cell line).

Supplementary tables

Supplementary table 1: Enriched pathways in neuroblastoma cohorts. Pathway enrichment for NB1 (E-MTAB-1781) and NB2 cohorts (TARGET). GSEA pathway enrichment in MYCN correlated gene list and MNA versus non-MNA gene list. In the table are listed the first 100 pathways.

Supplementary table 2: Enriched pathways in MYCN correlating gene list. GSEA Pathway enrichment in MYCN correlated gene list in different malignancies (NB1: E-MTAB-1781, NB2: TARGET neuroblastoma, Wilms: TARGET Wilms, SCLC1: E-MTAB-1999, SCLC2: PMID 26168399, RB: GSE59983, Rhabdo: GSE114621, T-ALL: GSE13159, AML: GSE13159). In the table are listed the first 100 pathways.

Supplementary table 3: Enriched pathways in MYC correlating gene list. GSEA Pathway enrichment in MYC correlated gene list in different malignancies (NB1: E-MTAB-1781, NB2: TARGET neuroblastoma, Wilms: TARGET Wilms, SCLC1: E-MTAB-1999, SCLC2: PMID 26168399, RB: GSE59983, Rhabdo: GSE114621, T-ALL: GSE13159, AML: GSE13159). In the table are listed the first 100 pathways.

Supplementary table 4: WGCNA module assigned genes. Module 0 represents genes not assigned to any modules. Module column (first column) indicate the WGCNA modules.

Supplementary table 5: WGCNA module pathway enrichment. GO pathway enriched in the WGCNA module. Module column (first column) indicate the WGCNA modules.

Supplementary table 6: List of the 50 regulons in the NB1 cohort. (NB1: E-MTAB-1781)

Supplementary table 7: List of the 50 regulons in the NB2 cohort. (NB2: TARGET neuroblastoma)

Supplementary table 8: List of the first 20 pathways enriched in each regulon cluster.

Supplementary table 9: List of genes used for the logistic regression model.

Supplementary table 10: List of significant genes: univariate Cox regression. Only significantly genes are present

Supplementary table 10: List of significant genes: univariate Cox regression

Supplementary table 11: List of genes and corresponding weights in the logistic regression model.

Supplementary table 12: clinical information for neuroblastoma dataset (accession: E-MTAB-1781). MYCN_status columns represent amplification status (MNA for MYCN amplification, non-MNA for not amplified), sex column, Age (in days), OS_d represents overall survivals, OS_bin (Overall surviva status, 1 death, 0 censored), Stage (2A and 2B collapsed as 2), imm_z (MYCN immune score normalized, z-score), MYCN immuno signature (MYCN immune status group stratification)

Supplementary table 13: clinical information for neuroblastoma dataset (accession: TARGET). MYCN_status columns represent amplification status (MNA for MYCN amplification, non-MNA for not

amplified), Gender, Age represents age at the diagnosis (in days), OS_d represents overall survivals, OS_bin (Overall surviva status, 1 death, 0 censored), Stage, imm_z (MYCN immune score normalized, z-score), MYCN immuno signature (MYCN immune status group stratification)

Supplementary table 14: list of cell lines used in this study. For each cell line is listed the source, the MYCN-status, source, data obtained, mycoplasma testing, citation.

Supplementary table 15: List of primers used in this study. Sense and anti-sense

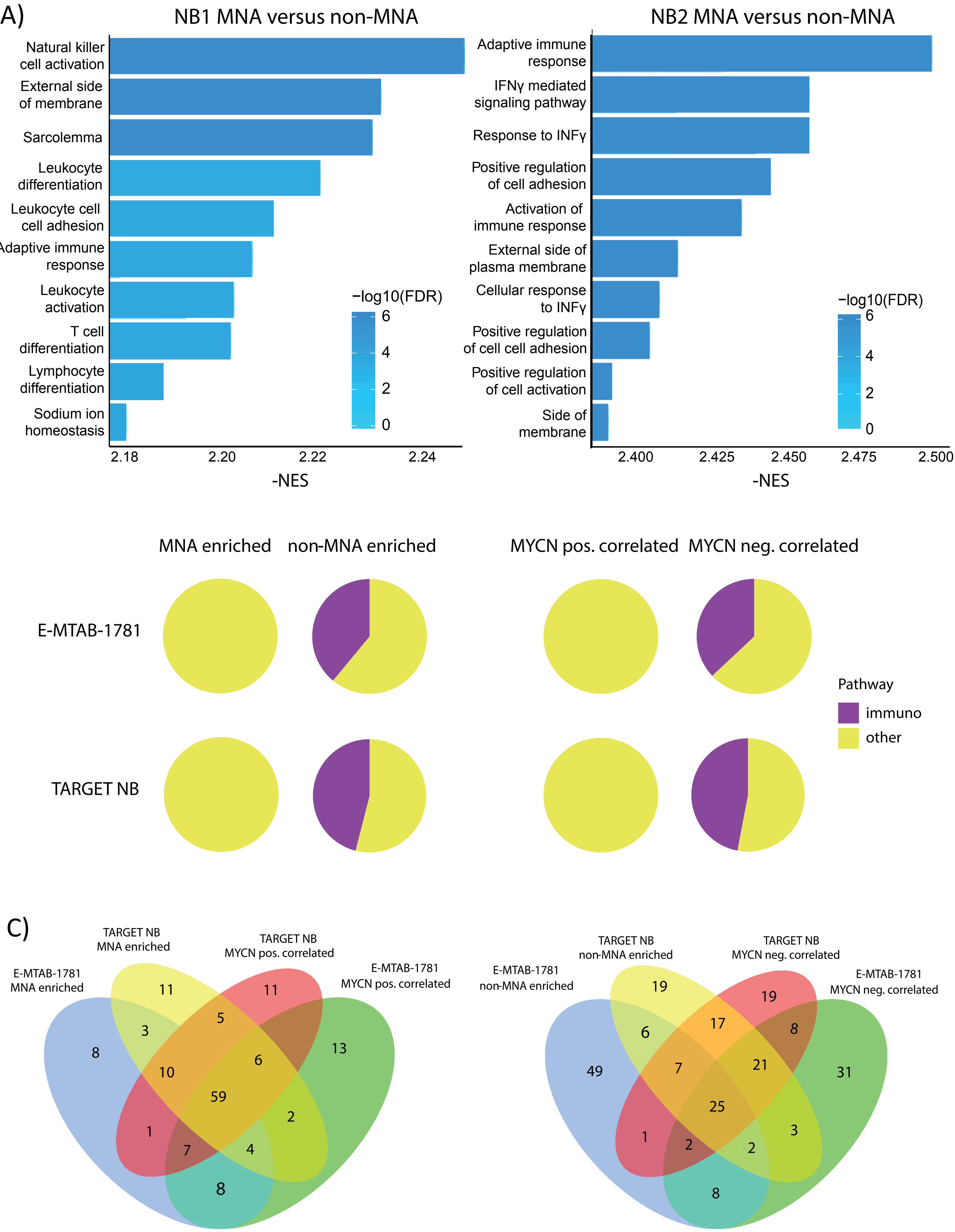

A)
NB1 MNA versus non-MNA
Natural killer cell activation
External side of membrane
Sarcolemma
Leukocyte differentiation
Leukocyte cell cell adhesion
Adaptive immune response
Leukocyte activation
T cell differentiation
Lymphocyte differentiation
Sodium ion homeostasis
−log10(FDR)
6
4
2
0
2.18
2.20
2.22
2.24
-NES
NB2 MNA versus non-MNA
Adaptive immune response
IFNγ mediated signaling pathway
Response to INFγ
Positive regulation of cell adhesion
Activation of immune response
External side of plasma membrane
Cellular response to INFγ
Positive regulation of cell cell adhesion
Positive regulation of cell activation
Side of membrane
−log10(FDR)
6
4
2
0
2.400
2.425
2.450
2.475
2.500
-NES
MNA enriched
non-MNA enriched
MYCN pos. correlated
MYCN neg. correlated
E-MTAB-1781
TARGET NB
Pathway
immuno
other
C)
TARGET NB MNA enriched
TARGET NB MYCN pos. correlated
E-MTAB-1781 MNA enriched
E-MTAB-1781 MYCN pos. correlated
11
11
3
5
8
13
10
6
59
2
1
7
4
8
TARGET NB non-MNA enriched
TARGET NB MYCN neg. correlated
E-MTAB-1781 non-MNA enriched
E-MTAB-1781 MYCN neg. correlated
19
19
6
17
8
49
7
21
31
25
1
2
3
2
8

A)

Dataset	Cancer	MYCN corr. Pathway	Immune pathway	Other
**E-MTAB-1781**	**Neuroblastoma**	pos. enrich.	0	100
		neg. enrich.	37	63
**TARGET**	**Neuroblastoma**	pos. enrich.	0	100
		neg. enrich.	47	53
TARGET	Wilms	pos. enrich.	0	100
		neg. enrich.	11	89
GSE 59983	Retinoblastoma	pos. enrich.	0	100
		neg. enrich.	9	91
GSE 114621	Rhabdomyosarcoma	pos. enrich.	0	100
		neg. enrich.	46	54
GSE 13159	T-ALL	pos. enrich.	5	95
		neg. enrich.	16	84
GSE 13159	AML	pos. enrich.	0	100
		neg. enrich.	29	71
PMID: 26168399	SCLC	pos. enrich.	0	100
		neg. enrich.	61	39
E-MTAB-1999	SCLC	pos. enrich.	0	100
		neg. enrich.	15	85

B)

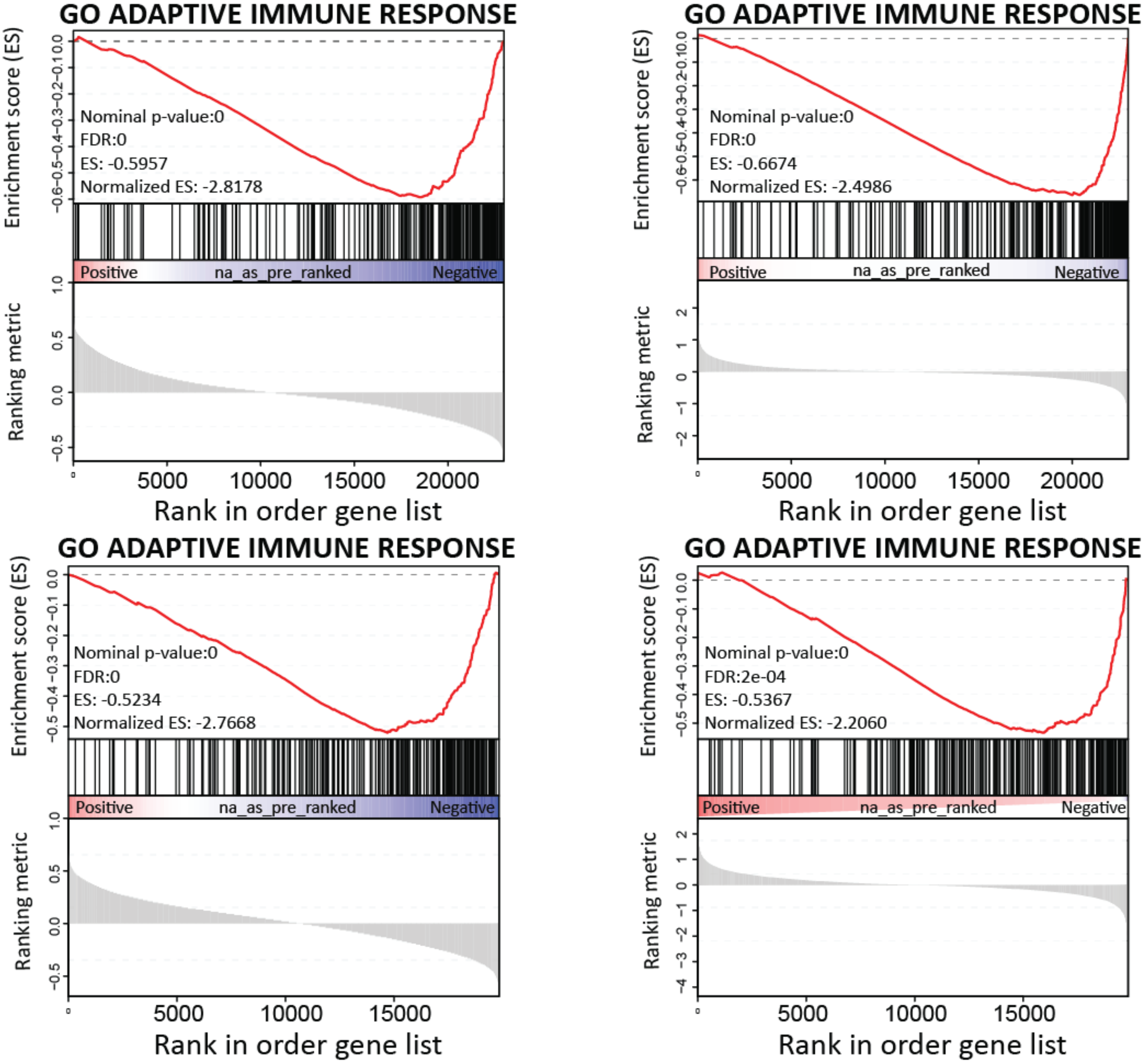

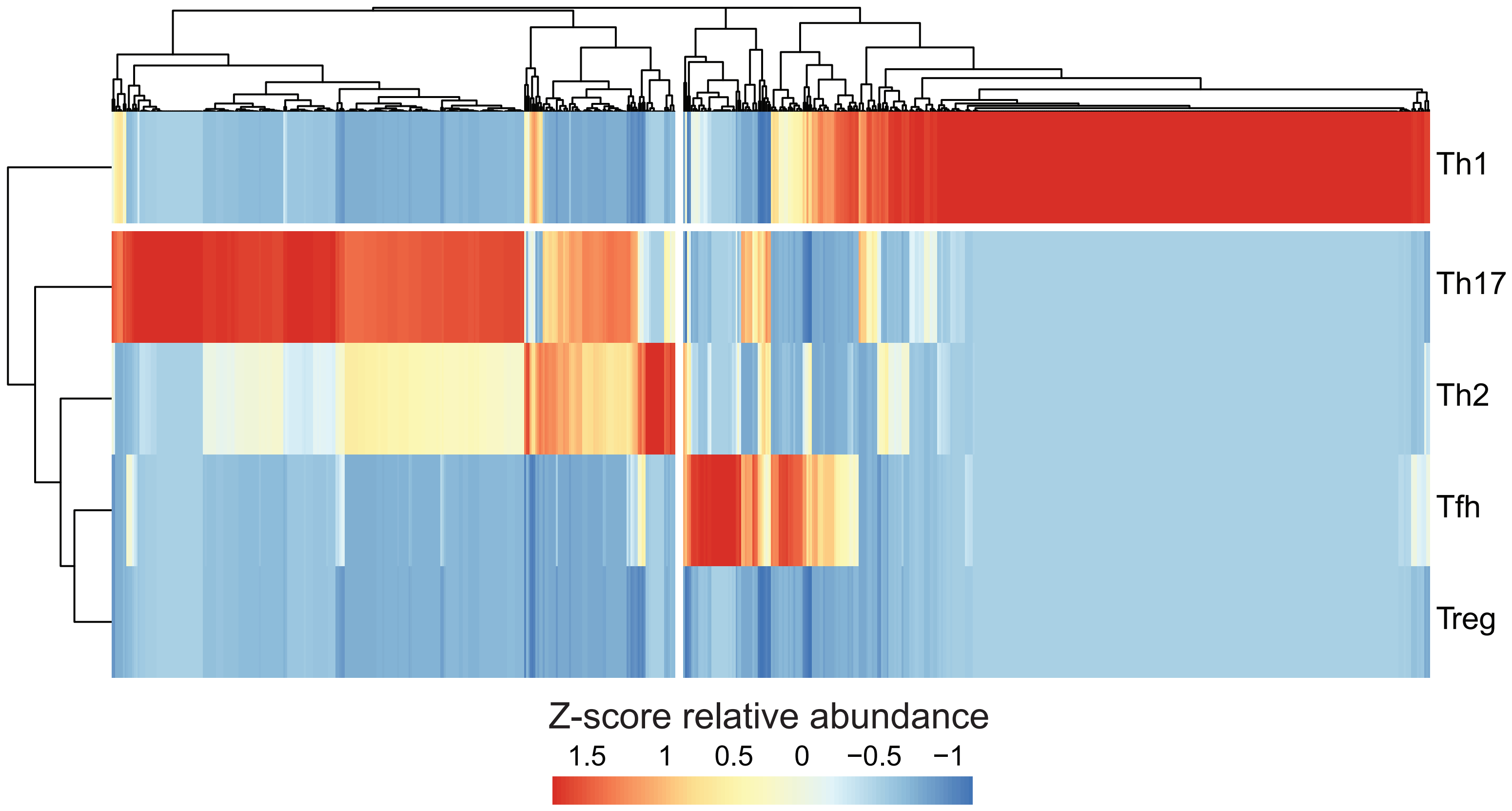
Th1
Th17
Th2
Tfh
Treg
Z-score relative abundance
1.5
1
0.5
0
−0.5
−1

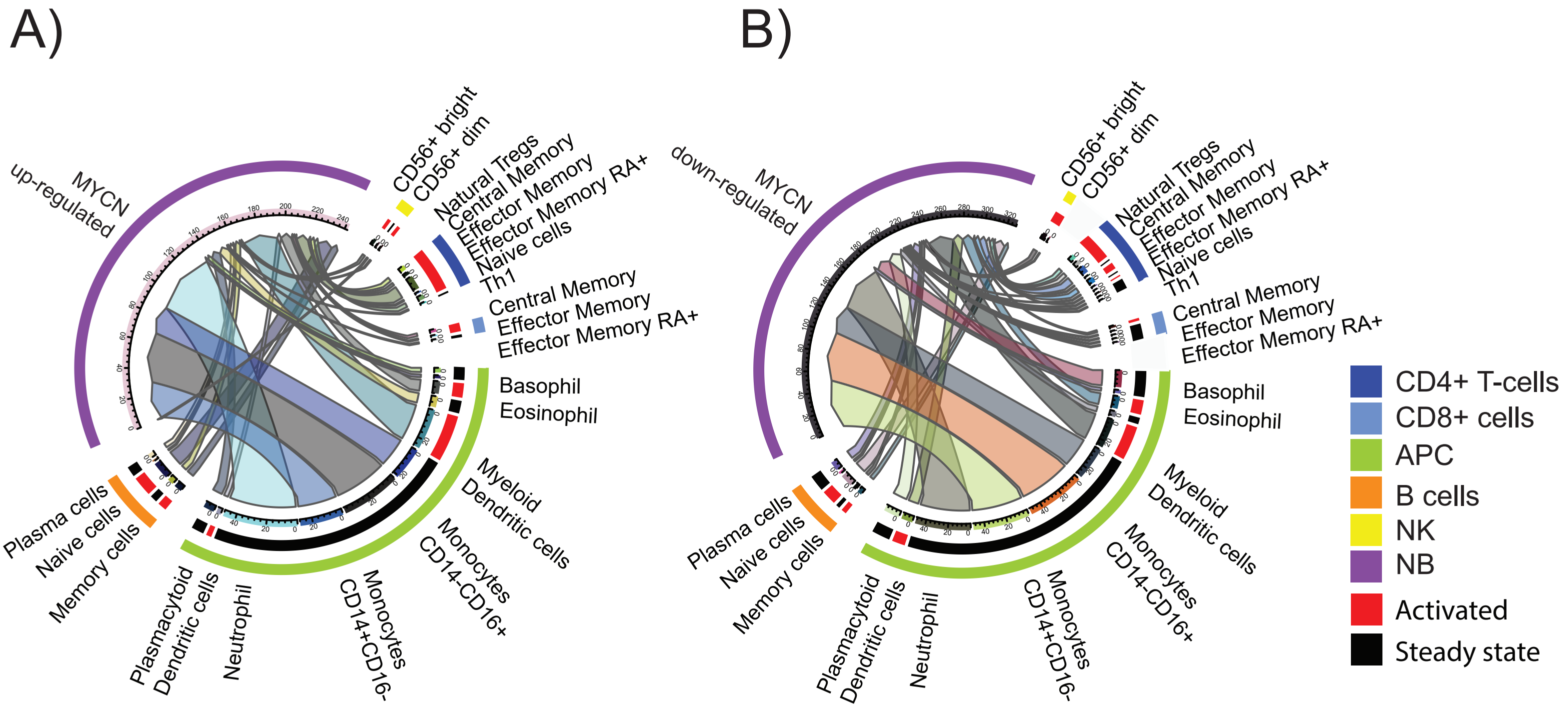
A)
B)
MYCN up-regulated
MYCN down-regulated
CD56+ bright
CD56+ dim
Natural Tregs
Central Memory
Effector Memory
Effector Memory RA+
Naive cells
Th1
Central Memory
Effector Memory
Effector Memory RA+
Basophil
Eosinophil
Myeloid Dendritic cells
Monocytes CD14-CD16+
Monocytes CD14+CD16-
Neutrophil
Plasmacytoid Dendritic cells
Plasma cells
Naive cells
Memory cells
CD4+ T-cells
CD8+ cells
APC
B cells
NK
NB
Activated
Steady state

A)

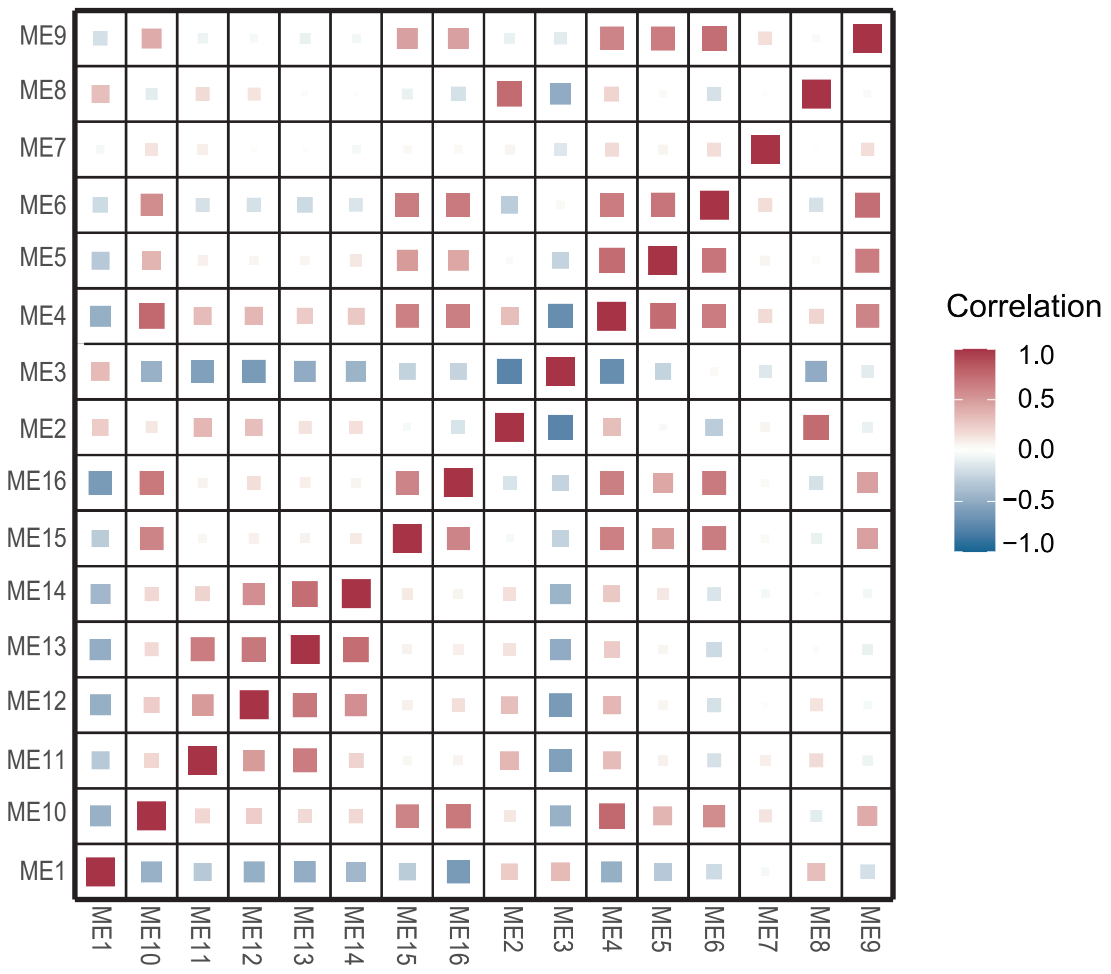
ME9
ME8
ME7
ME6
ME5
ME4
ME3
ME2
ME16
ME15
ME14
ME13
ME12
ME11
ME10
ME1
ME1
ME10
ME11
ME12
ME13
ME14
ME15
ME16
ME2
ME3
ME4
ME5
ME6
ME7
ME8
ME9
Correlation
1.0
0.5
0.0
−0.5
−1.0

B)

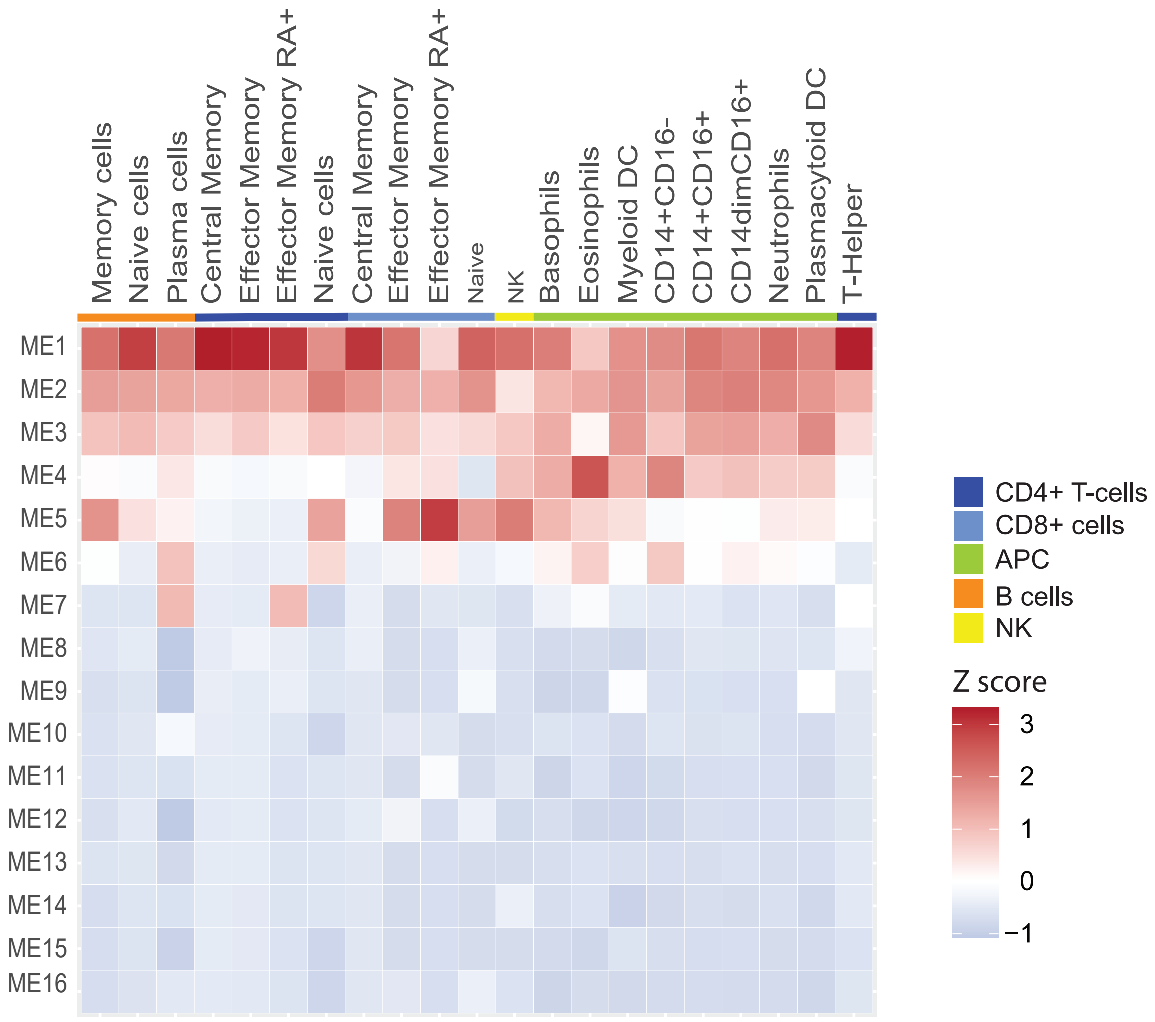
Memory cells
Naive cells
Plasma cells
Central Memory
Effector Memory
Effector Memory RA+
Naive cells
Central Memory
Effector Memory
Effector Memory RA+
Naive
NK
Basophils
Eosinophils
Myeloid DC
CD14+CD16-
CD14+CD16+
CD14dimCD16+
Neutrophils
Plasmacytoid DC
T-Helper
ME1
ME2
ME3
ME4
ME5
ME6
ME7
ME8
ME9
ME10
ME11
ME12
ME13
ME14
ME15
ME16
CD4+ T-cells
CD8+ cells
APC
B cells
NK
Z score
3
2
1
0
−1

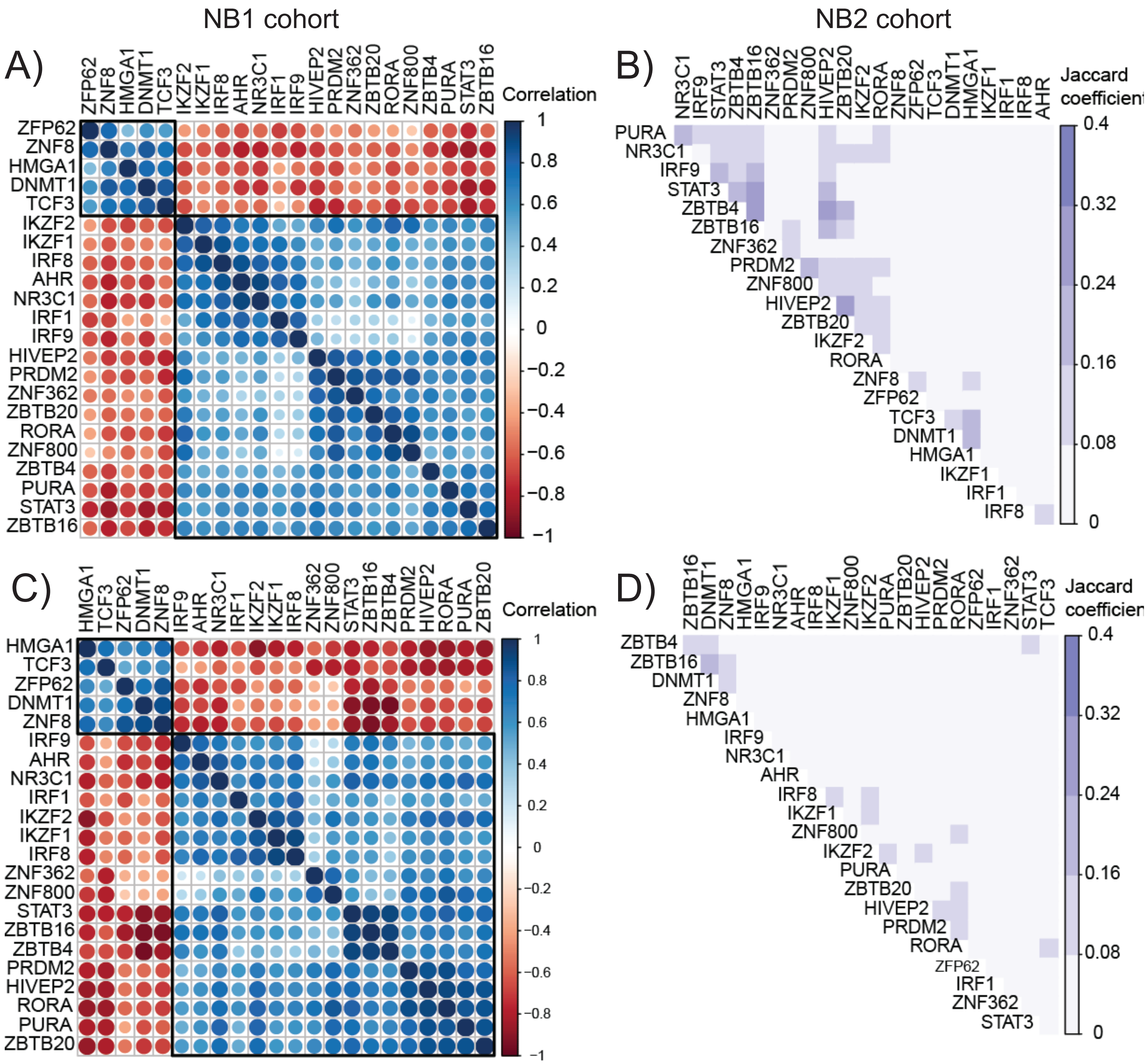
NB1 cohort
NB2 cohort
A)
ZFP62 ZNF8 HMGA1 DNMT1 TCF3 IKZF2 IKZF1 IRF8 AHR NR3C1 IRF1 IRF9 HIVEP2 PRDM2 ZNF362 ZBTB20 RORA ZNF800 ZBTB4 PURA STAT3 ZBTB16
Correlation
1 0.8 0.6 0.4 0.2 0 −0.2 −0.4 −0.6 −0.8 −1
B)
NR3C1 IRF9 STAT3 ZBTB4 ZBTB16 ZNF362 PRDM2 ZNF800 HIVEP2 ZBTB20 IKZF2 RORA ZNF8 ZFP62 TCF3 DNMT1 HMGA1 IKZF1 IRF1 IRF8 AHR
PURA NR3C1 IRF9 STAT3 ZBTB4 ZBTB16 ZNF362 PRDM2 ZNF800 HIVEP2 ZBTB20 IKZF2 RORA ZNF8 ZFP62 TCF3 DNMT1 HMGA1 IKZF1 IRF1 IRF8
Jaccard coefficien
0.4 0.32 0.24 0.16 0.08 0
C)
HMGA1 TCF3 ZFP62 DNMT1 ZNF8 IRF9 AHR NR3C1 IRF1 IKZF2 IKZF1 IRF8 ZNF362 ZNF800 STAT3 ZBTB16 ZBTB4 PRDM2 HIVEP2 RORA PURA ZBTB20
Correlation
1 0.8 0.6 0.4 0.2 0 −0.2 −0.4 −0.6 −0.8 −1
D)
ZBTB16 DNMT1 ZNF8 HMGA1 IRF9 NR3C1 AHR IRF8 IKZF1 ZNF800 IKZF2 PURA ZBTB20 HIVEP2 PRDM2 RORA ZFP62 IRF1 ZNF362 STAT3 TCF3
ZBTB4 ZBTB16 DNMT1 ZNF8 HMGA1 IRF9 NR3C1 AHR IRF8 IKZF1 ZNF800 IKZF2 PURA ZBTB20 HIVEP2 PRDM2 RORA ZFP62 IRF1 ZNF362 STAT3
Jaccard coefficien
0.4 0.32 0.24 0.16 0.08 0

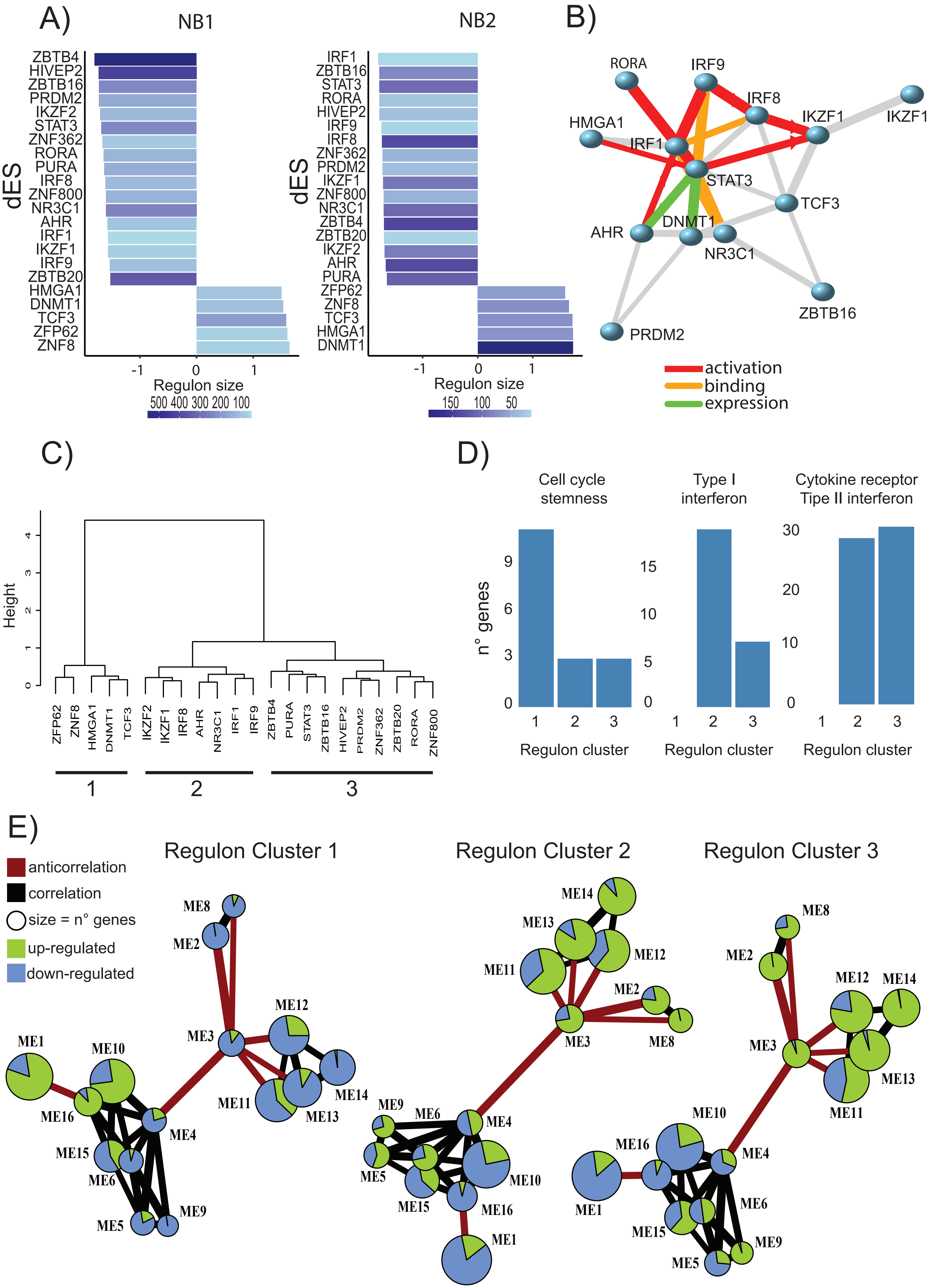

A)
NB1
NB2
dES
ZBTB4
HIVEP2
ZBTB16
PRDM2
IKZF2
STAT3
ZNF362
RORA
PURA
IRF8
ZNF800
NR3C1
AHR
IRF1
IKZF1
IRF9
ZBTB20
HMGA1
DNMT1
TCF3
ZFP62
ZNF8
Regulon size
500 400 300 200 100
150 100 50
B)
RORA
IRF9
IRF8
IKZF1
HMGA1
IRF1
STAT3
TCF3
AHR
DNMT1
NR3C1
ZBTB16
PRDM2
activation
binding
expression
C)
Height
1
2
3
D)
Cell cycle stemness
Type I interferon
Cytokine receptor Tipe II interferon
n° genes
Regulon cluster
E)
Regulon Cluster 1
Regulon Cluster 2
Regulon Cluster 3
anticorrelation
correlation
size = n° genes
up-regulated
down-regulated

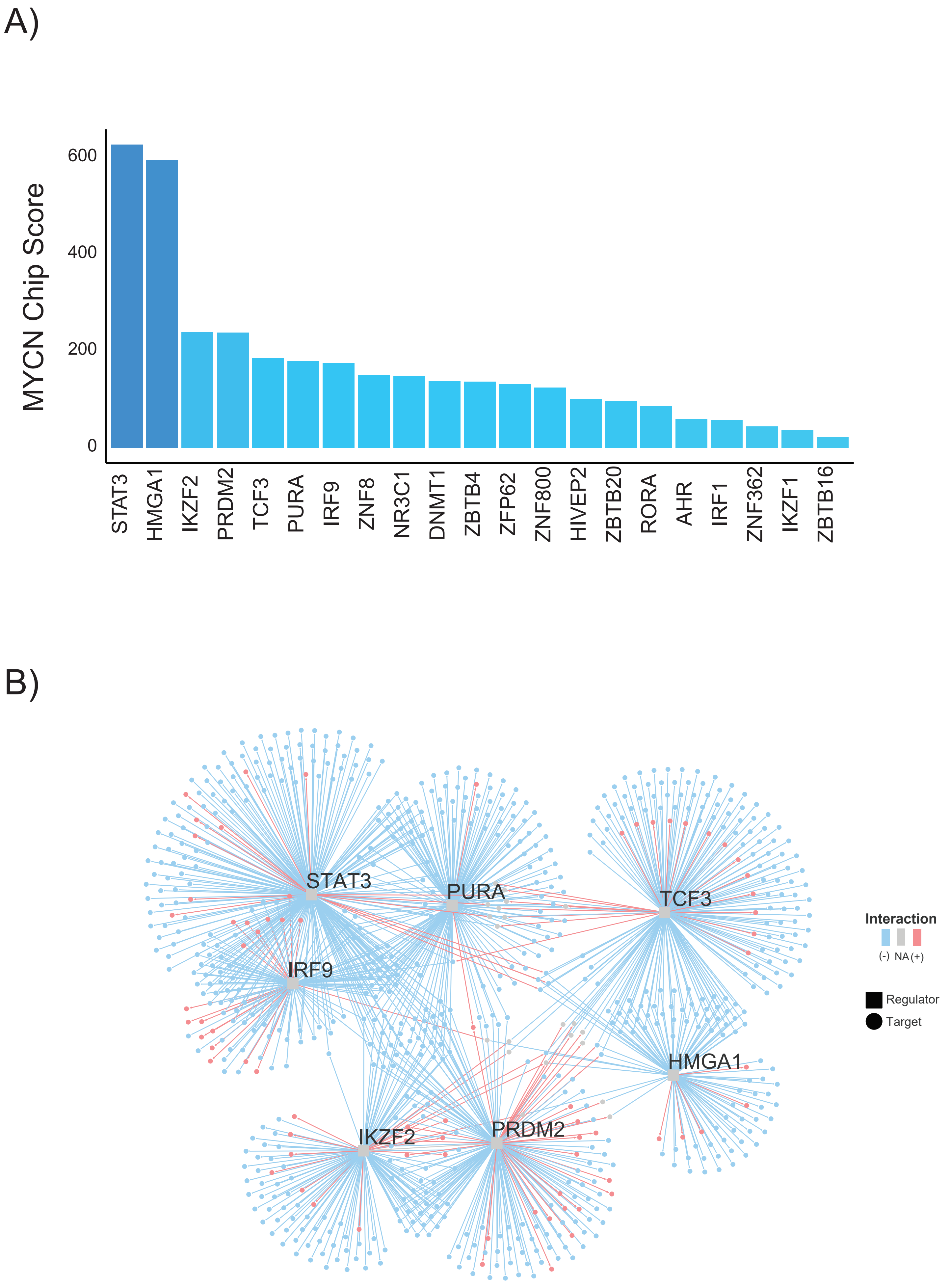
A)
MYCN Chip Score
600
400
200
0
STAT3
HMGA1
IKZF2
PRDM2
TCF3
PURA
IRF9
ZNF8
NR3C1
DNMT1
ZBTB4
ZFP62
ZNF800
HIVEP2
ZBTB20
RORA
AHR
IRF1
ZNF362
IKZF1
ZBTB16
B)
STAT3
PURA
TCF3
IRF9
HMGA1
IKZF2
PRDM2
Interaction
(-)
NA
(+)
Regulator
Target

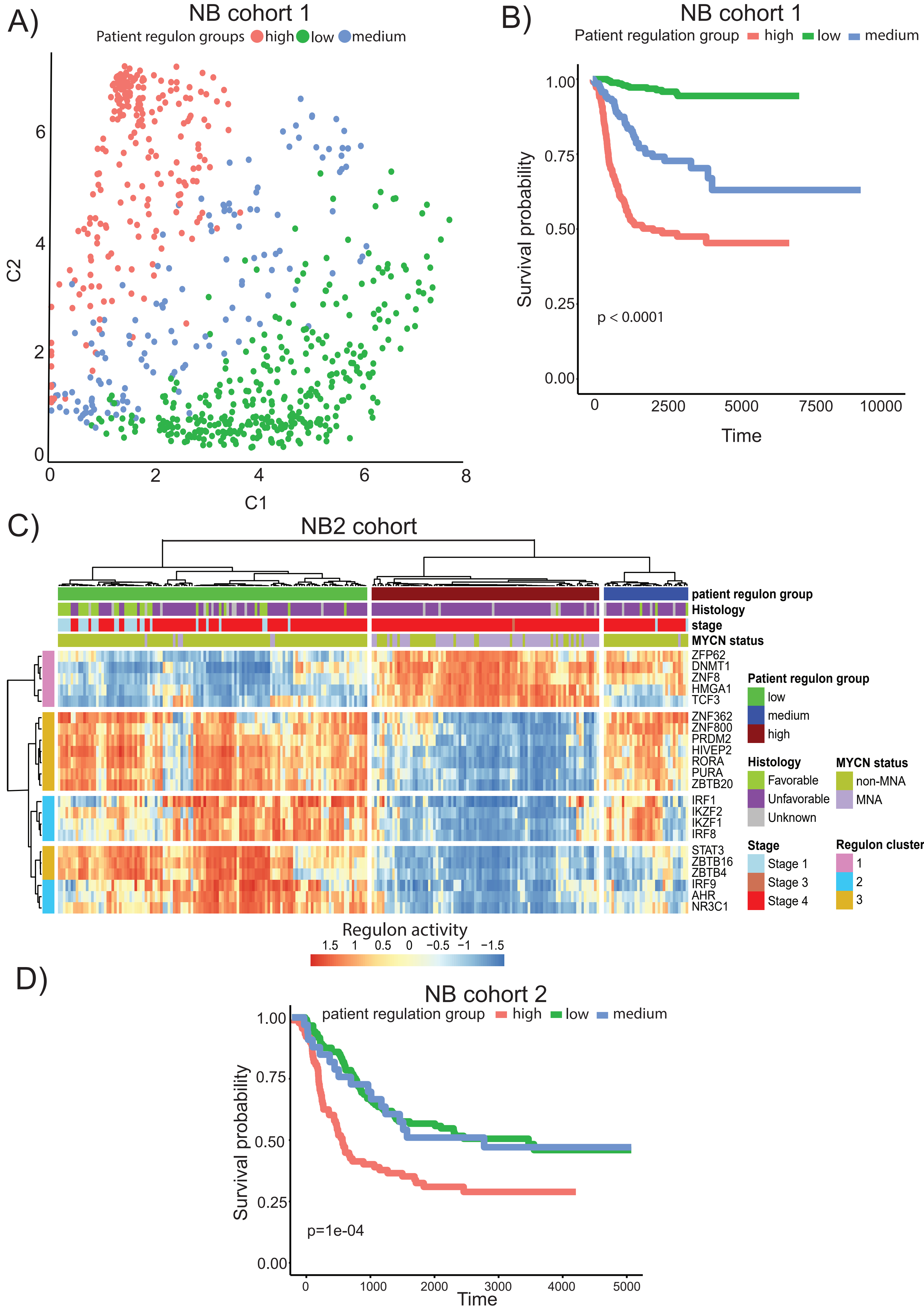
A)
NB cohort 1
Patient regulon groups high low medium
C2
C1
B)
NB cohort 1
Patient regulation group high low medium
Survival probability
p < 0.0001
Time
C)
NB2 cohort
patient regulon group
Histology
stage
MYCN status
ZFP62
DNMT1
ZNF8
HMGA1
TCF3
ZNF362
ZNF800
PRDM2
HIVEP2
RORA
PURA
ZBTB20
IRF1
IKZF2
IKZF1
IRF8
STAT3
ZBTB16
ZBTB4
IRF9
AHR
NR3C1
Patient regulon group
low
medium
high
Histology
Favorable
Unfavorable
Unknown
MYCN status
non-MNA
MNA
Stage
Stage 1
Stage 3
Stage 4
Regulon cluster
1
2
3
Regulon activity
1.5 1 0.5 0 −0.5 −1 −1.5
D)
NB cohort 2
patient regulation group high low medium
Survival probability
p=1e-04
Time

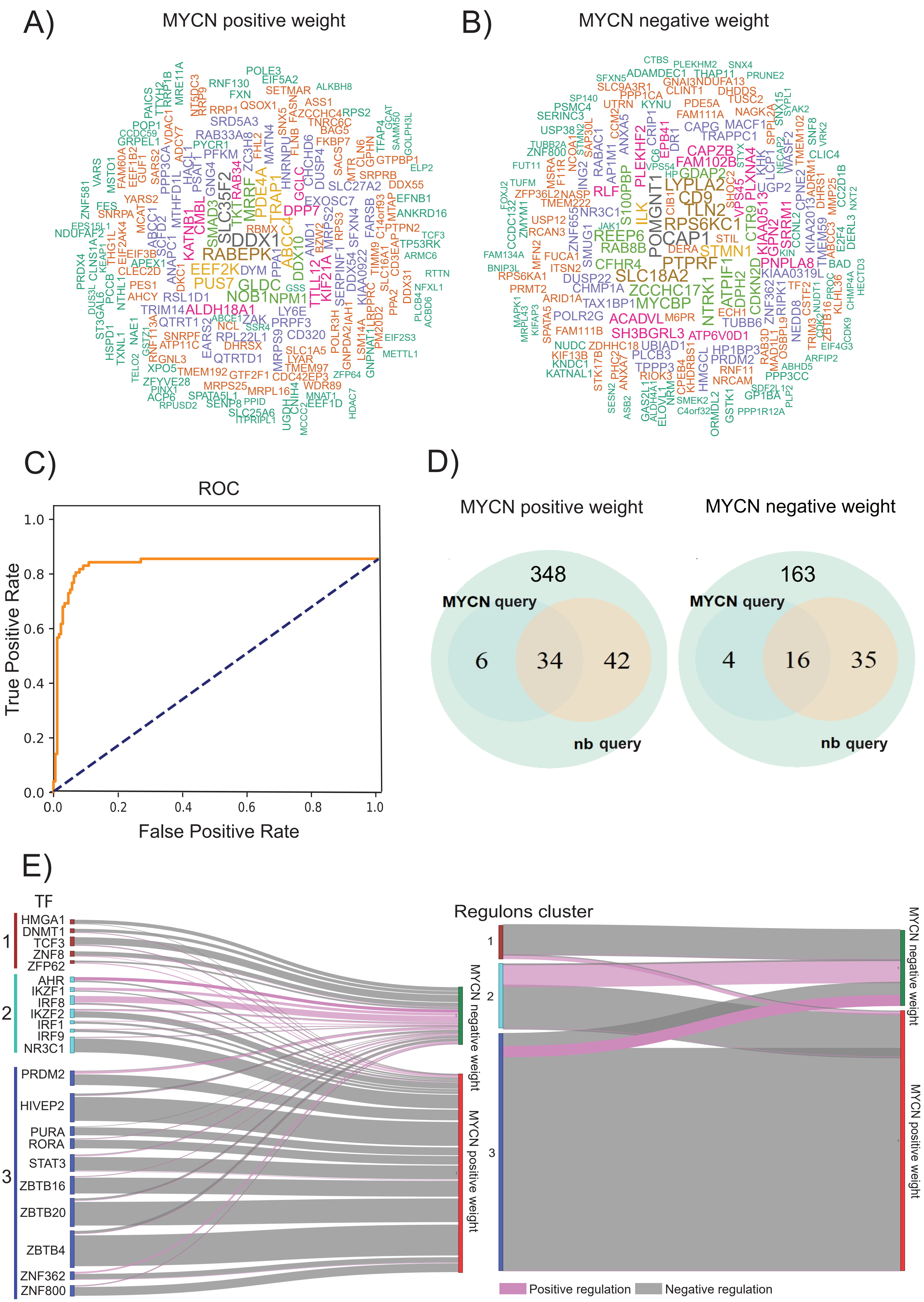

A)
MYCN positive weight
B)
MYCN negative weight
C)
ROC
True Positive Rate
False Positive Rate
0.0
0.2
0.4
0.6
0.8
1.0
D)
MYCN positive weight
MYCN negative weight
348
163
MYCN query
nb query
6
34
42
4
16
35
E)
TF
HMGA1
DNMT1
TCF3
ZNF8
ZFP62
AHR
IKZF1
IRF8
IKZF2
IRF1
IRF9
NR3C1
PRDM2
HIVEP2
PURA
RORA
STAT3
ZBTB16
ZBTB20
ZBTB4
ZNF362
ZNF800
1
2
3
MYCN negative weight
MYCN positive weight
Regulons cluster
Positive regulation
Negative regulation

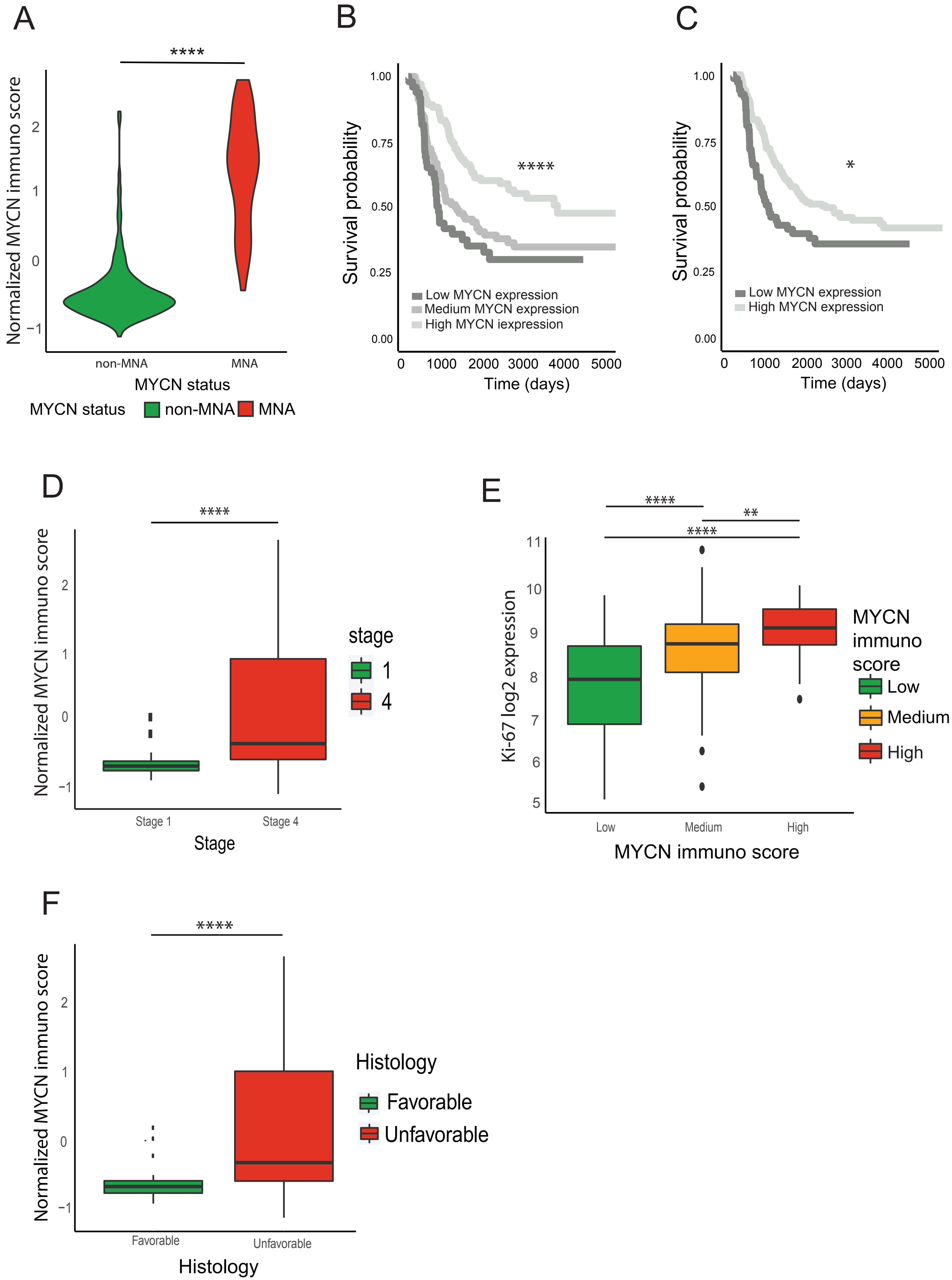
A
****
Normalized MYCN immuno score
2
1
0
−1
non-MNA
MNA
MYCN status
MYCN status
non-MNA
MNA
B
1.00
0.75
0.50
0.25
0.00
Survival probability
****
Low MYCN expression
Medium MYCN expression
High MYCN iexpression
0
1000
2000
3000
4000
5000
Time (days)
C
1.00
0.75
0.50
0.25
0.00
Survival probability
*
Low MYCN expression
High MYCN expression
0
1000
2000
3000
4000
5000
Time (days)
D
****
Normalized MYCN immuno score
2
1
0
−1
Stage 1
Stage 4
Stage
stage
1
4
E
****
**
****
11
10
9
8
7
6
5
Ki-67 log2 expression
Low
Medium
High
MYCN immuno score
MYCN immuno score
Low
Medium
High
F
****
Normalized MYCN immuno score
2
1
0
−1
Favorable
Unfavorable
Histology
Histology
Favorable
Unfavorable

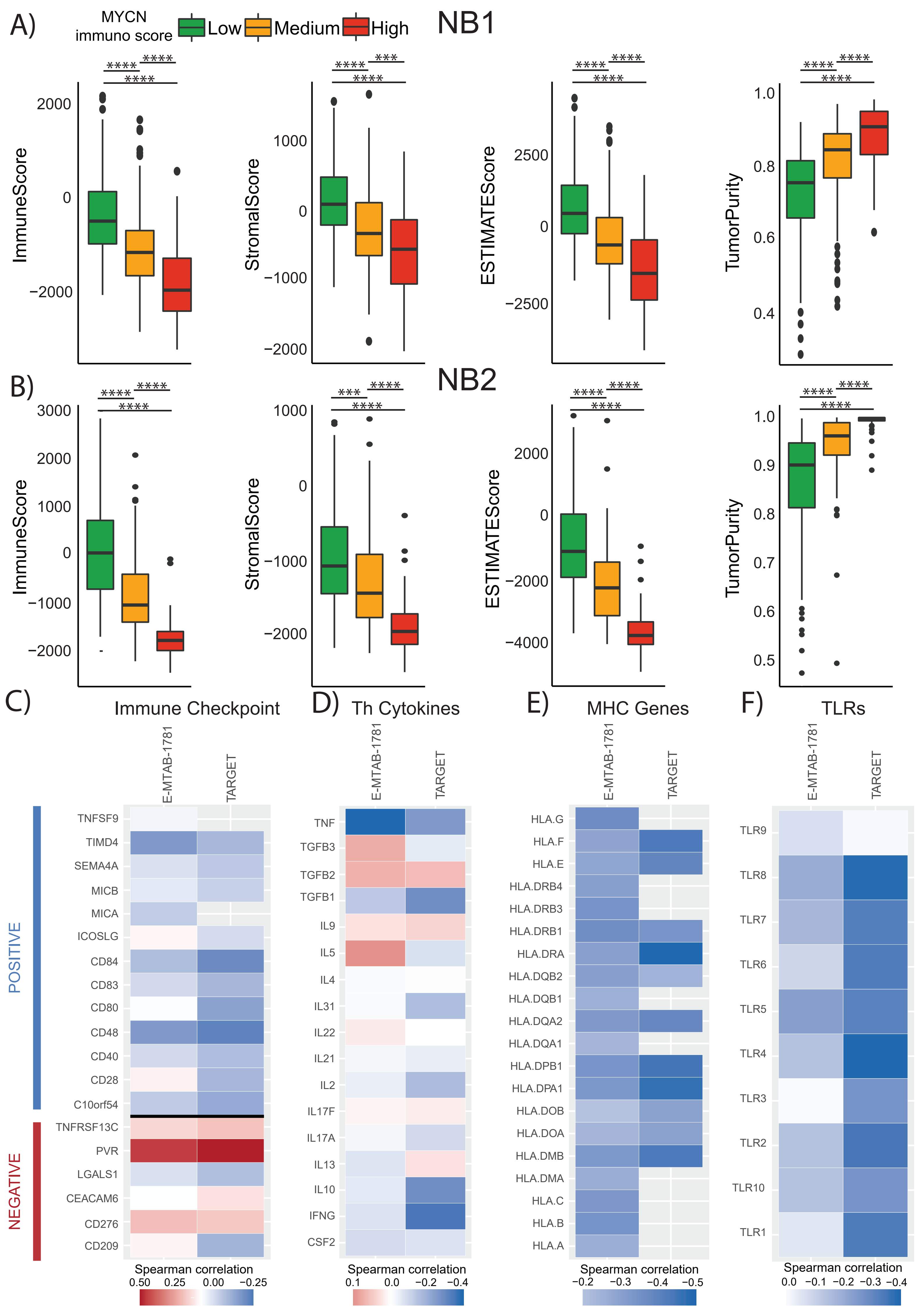

A)
MYCN immuno score
Low
Medium
High
NB1
ImmuneScore
StromalScore
ESTIMATEScore
TumorPurity
B)
NB2
C)
Immune Checkpoint
D)
Th Cytokines
E)
MHC Genes
F)
TLRs
E-MTAB-1781
TARGET
POSITIVE
NEGATIVE
TNFSF9
TIMD4
SEMA4A
MICB
MICA
ICOSLG
CD84
CD83
CD80
CD48
CD40
CD28
C10orf54
TNFRSF13C
PVR
LGALS1
CEACAM6
CD276
CD209
TNF
TGFB3
TGFB2
TGFB1
IL9
IL5
IL4
IL31
IL22
IL21
IL2
IL17F
IL17A
IL13
IL10
IFNG
CSF2
HLA.G
HLA.F
HLA.E
HLA.DRB4
HLA.DRB3
HLA.DRB1
HLA.DRA
HLA.DQB2
HLA.DQB1
HLA.DQA2
HLA.DQA1
HLA.DPB1
HLA.DPA1
HLA.DOB
HLA.DOA
HLA.DMB
HLA.DMA
HLA.C
HLA.B
HLA.A
TLR9
TLR8
TLR7
TLR6
TLR5
TLR4
TLR3
TLR2
TLR10
TLR1
Spearman correlation

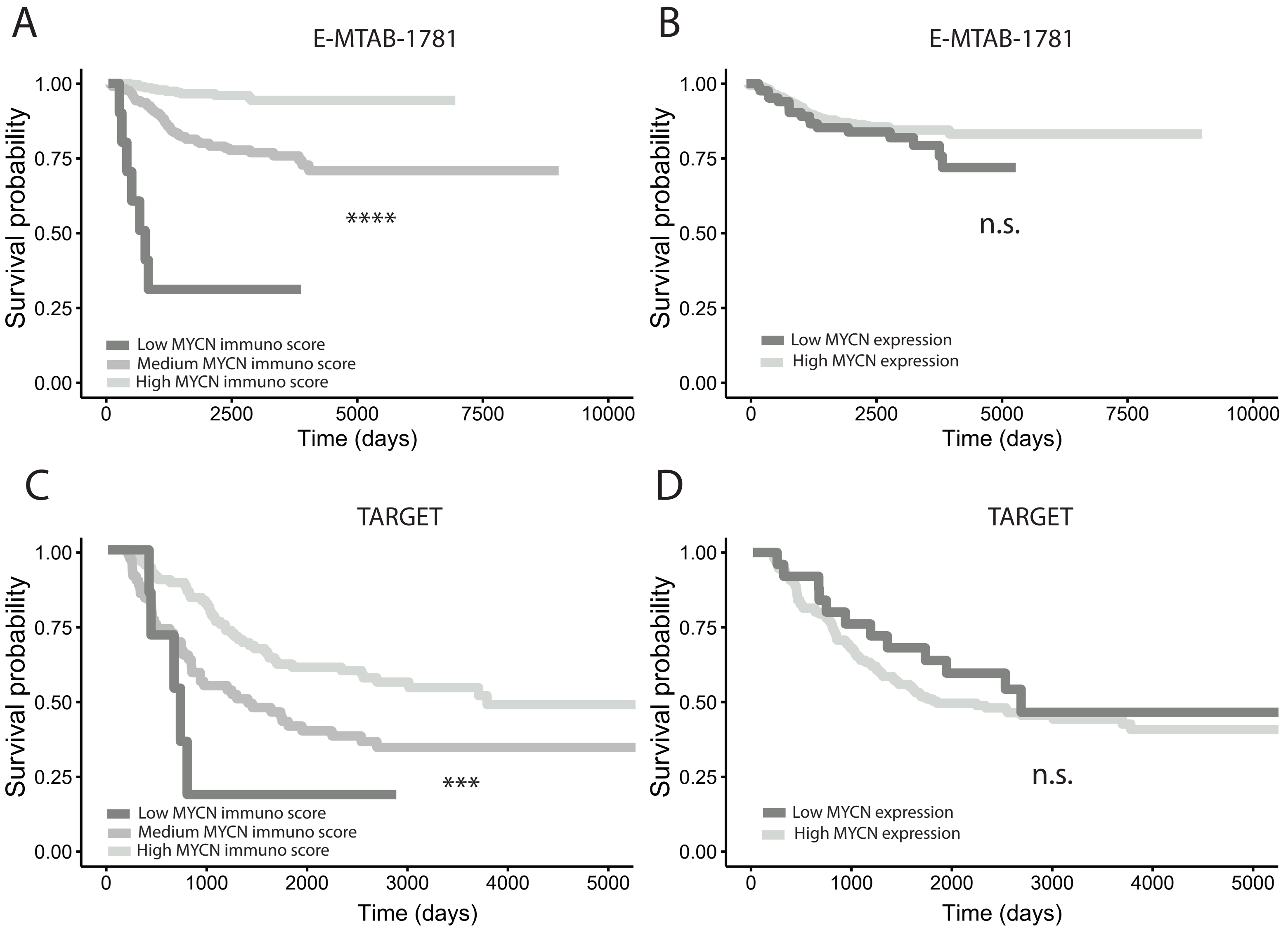
A
E-MTAB-1781
Survival probability
1.00
0.75
0.50
0.25
0.00
****
Low MYCN immuno score
Medium MYCN immuno score
High MYCN immuno score
0
2500
5000
7500
10000
Time (days)
B
E-MTAB-1781
n.s.
Low MYCN expression
High MYCN expression
C
TARGET
***
0
1000
2000
3000
4000
5000
D
TARGET
n.s.

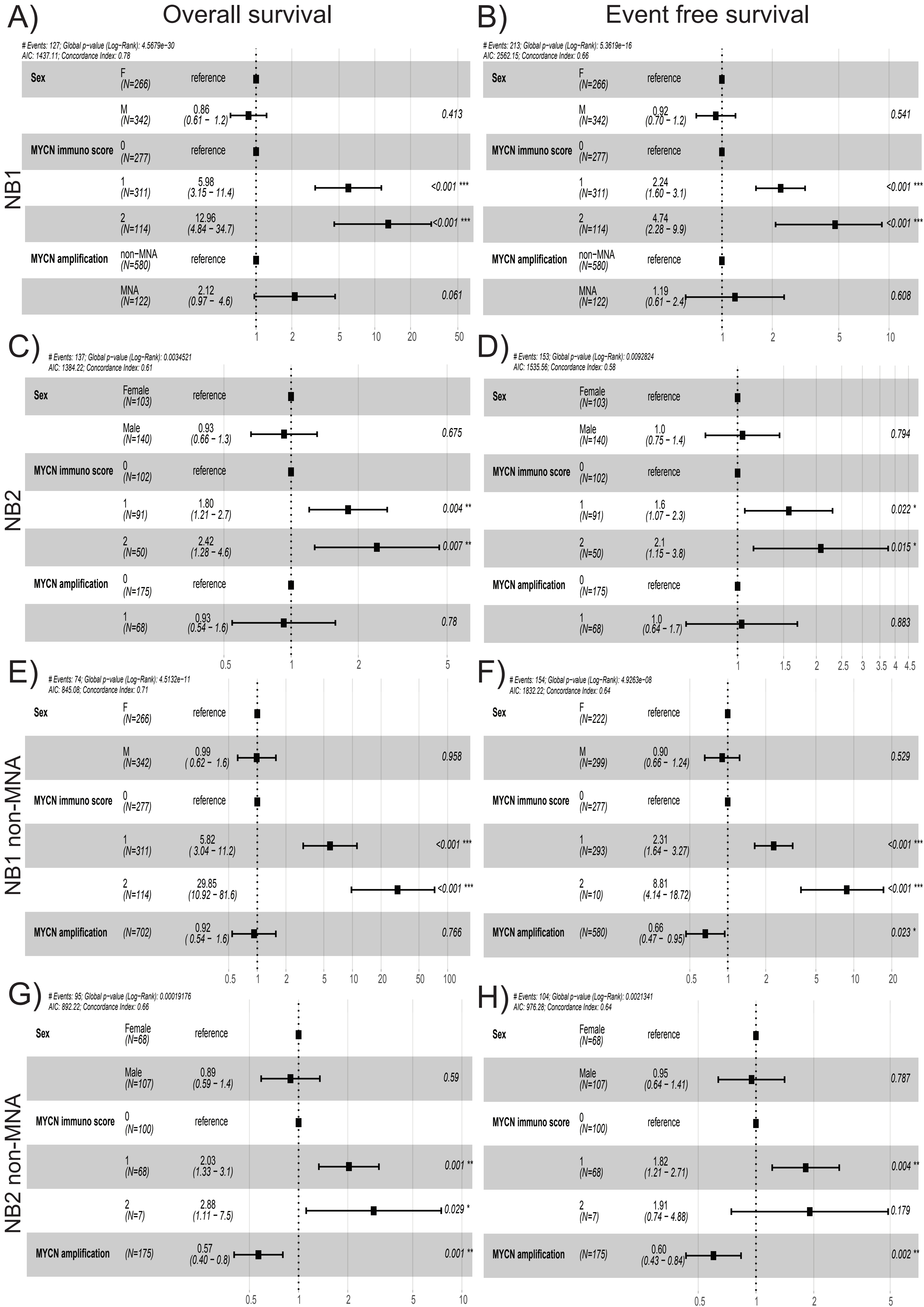

A)
Overall survival
B)
Event free survival
C)
D)
E)
F)
G)
H)
NB1
NB2
NB1 non-MNA
NB2 non-MNA
Events: 127; Global p-value (Log-Rank): 4.5679e−30
AIC: 1437.11; Concordance Index: 0.78
Events: 213; Global p-value (Log-Rank): 5.3619e−16
AIC: 2562.15; Concordance Index: 0.66
Events: 137; Global p-value (Log-Rank): 0.0034521
AIC: 1384.22; Concordance Index: 0.61
Events: 153; Global p-value (Log-Rank): 0.0092824
AIC: 1535.56; Concordance Index: 0.58
Events: 74; Global p-value (Log-Rank): 4.5132e−11
AIC: 845.08; Concordance Index: 0.71
Events: 154; Global p-value (Log-Rank): 4.9263e−08
AIC: 1832.22; Concordance Index: 0.64
Events: 95; Global p-value (Log-Rank): 0.00019176
AIC: 892.22; Concordance Index: 0.66
Events: 104; Global p-value (Log-Rank): 0.0021341
AIC: 976.28; Concordance Index: 0.64
Sex
MYCN immuno score
MYCN amplification

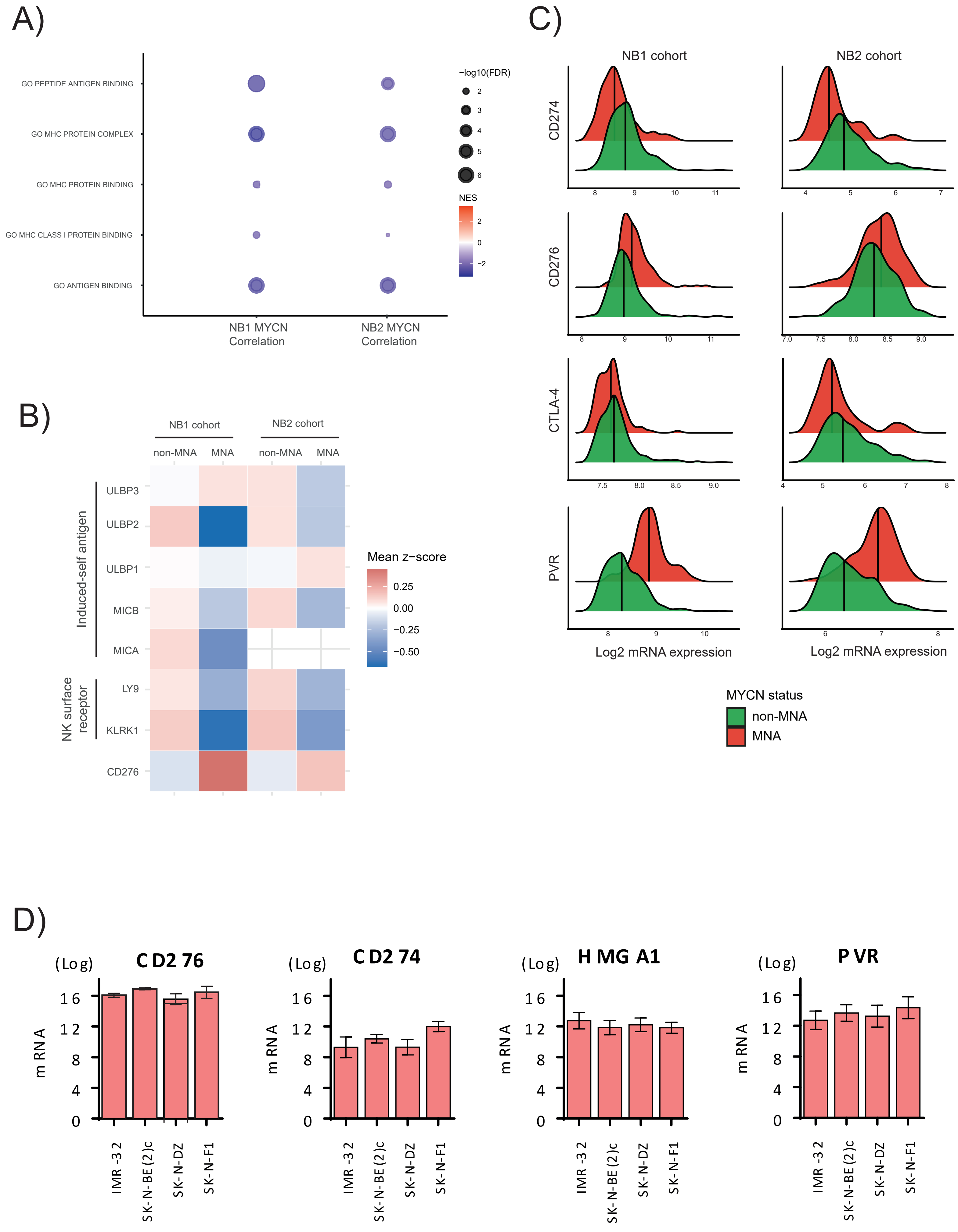
A)
GO PEPTIDE ANTIGEN BINDING
GO MHC PROTEIN COMPLEX
GO MHC PROTEIN BINDING
GO MHC CLASS I PROTEIN BINDING
GO ANTIGEN BINDING
NB1 MYCN Correlation
NB2 MYCN Correlation
−log10(FDR)
2
3
4
5
6
NES
2
0
−2
B)
NB1 cohort
NB2 cohort
non-MNA
MNA
non-MNA
MNA
Induced-self antigen
ULBP3
ULBP2
ULBP1
MICB
MICA
NK surface receptor
LY9
KLRK1
CD276
Mean z−score
0.25
0.00
−0.25
−0.50
C)
NB1 cohort
NB2 cohort
CD274
CD276
CTLA-4
PVR
Log2 mRNA expression
Log2 mRNA expression
MYCN status
non-MNA
MNA
D)
(Log)
CD276
(Log)
CD274
(Log)
HMGA1
(Log)
PVR
mRNA
16
12
8
4
0
IMR-32
SK-N-BE(2)c
SK-N-DZ
SK-N-F1
